# Strategies to enhance risk communication about medicines in Malaysia: a Delphi study among multinational experts

**DOI:** 10.1186/s12913-024-11476-0

**Published:** 2024-09-03

**Authors:** Rema Panickar, Zoriah Aziz, Chin Hai Teo, Adeeba Kamarulzaman

**Affiliations:** 1 Department of Medicine, Faculty of Medicine, Universiti Malaya, Kuala Lumpur, Malaysia; 2 National Pharmaceutical Regulatory Agency, Ministry of Health, Petaling Jaya, Malaysia; 3 Faculty of Medicine, Universiti Malaya, Kuala Lumpur, 50603 Malaysia; 4 Faculty of Pharmacy, MAHSA University, Bandar Saujana Putra, Malaysia; 5 Department of Primary Care Medicine, Faculty of Medicine, Universiti Malaya, Kuala Lumpur, Malaysia; 6 Monash University Malaysia, Subang Jaya, Malaysia

**Keywords:** Safety information, Effective communication, Pharmacovigilance, Developing country, Delphi survey, Consensus

## Abstract

**Background:**

Effective risk communication about medicines is crucial to the success of all pharmacovigilance activities but remains a worldwide challenge. Risk communication has been conducted in Malaysia for decades, yet awareness on the communication methods remains low among healthcare professionals. While international guidelines are available, clear guidance on effectively communicating the risks of medicines in specific countries is scarce. This study aimed to establish a consensus on the priority strategies for enhancing risk communication about medicines by regulators.

**Methods:**

We conducted a two-round modified Delphi survey among local and international communication experts, and also recipients of medicines risk communication in Malaysia. We developed a list of 37 strategies based on the findings of our previous studies. In Round 1, participants were asked to rate the priority for each strategy using a 5-point Likert scale and suggest additional strategies via free-text comments. Strategies scoring a mean of ≥ 3.75 were included in Round 2. We defined consensus for the final list of strategies a priori as > 75% agreement. Data were analysed using descriptive statistics and thematic analysis.

**Results:**

Our final Delphi panel (*n* = 39, 93% response rate) comprised medicines communication experts from nine countries and Malaysian healthcare professionals. Following Round 1, we dropped 14 strategies and added 11 strategies proposed by panellists. In the second round, 21 strategies achieved consensus. The priority areas identified were to improve the format and content of risk communication, increase the use of technology, and increase collaboration with various stakeholders. Priority ratings for the strategy “to offer incentives to pharmaceutical companies which maintain effective communication systems” were significantly higher among recipients compared to communicators [χ^2^_(1, *N* = 39)_ = 10.1; *p* = 0.039] and among local versus international panellists [χ^2^_(1, *N* = 39)_ = 14.3; *p* = 0.007].

**Conclusions:**

Our study identified 21 priority strategies, which were used to develop a strategic plan for enhancing medicines risk communication. This plan is potentially adaptable to all countries with developing pharmacovigilance systems. The difference in views between communicators and recipients, as well as local and international panellists, highlights the importance of involving multiple stakeholders in research.

**Supplementary Information:**

The online version contains supplementary material available at 10.1186/s12913-024-11476-0.

## Background

Medicinal products risk communication is an integral part of all pharmacovigilance processes [1]. Every medicine comes with benefits, but also carries the risk of adverse drug reactions. These risks need to be clearly communicated as part of pharmacovigilance risk minimisation activities to ensure the safe use of medicines. The importance of communication in pharmacovigilance has been discussed in international meetings since the 1990s [2, 3] and repeatedly highlighted in scientific literature [4–6]. Various measures have been taken to improve the effectiveness of communication, however, multiple challenges exist such as a lack of public trust in regulatory authorities, variable perceptions of risk depending on an individual’s knowledge, beliefs or experiences, health literacy gaps, and the more recent issues of misinformation or disinformation [1, 7, 8]. Thus, risk communication about medicines remains an important aspect of pharmacovigilance research.

Medicinal products are substances or combinations of substances intended to treat, prevent or diagnose a disease, or to restore, correct or modify physiological functions by exerting a pharmacological, immunological or metabolic action [9, 10]. Currently, there is no universally agreed definition for “medicinal product risk communication”. We have adapted the definition by Bahri [7], which describes the term as the structures, processes, and outcomes of information exchanges about risks and any concerns people may have with medicines, about the measures to support safe use and minimise risks and about risk governance overall in private, community and society spheres”. For simplicity, we use the term “medicines” to refer to “medicinal products”.

Many of the issues related to medicines risk communication may be considered global challenges, affecting almost all countries [8]. However, current research revealed that only a handful of countries have effective systems for communicating the risks associated with medicines [11]. For example among regulators, the European Medicines Agency (EMA) and the United States Food and Drug Administration have robust legislation on evaluating the effectiveness of risk communication, including requirements for the pharmaceutical industry to demonstrate the effectiveness of their risk communication. Bhasale et al. [11] reported that smaller regulators such as Health Canada and Therapeutics Goods Administration (TGA) Australia had legislation or regulations on risk communication but these did not fully describe the responsibilities of regulators and the industry. In contrast, NPRA Malaysia currently does not have legislation or guidelines specifically focused on risk communication [12]. While most countries face the same challenges in delivering effective risk communication about medicines, the difference lies in the resources, manpower and expertise available in each country to overcome these problems [13]. Several international bodies have released comprehensive guidelines on risk communication [14–16]. However the availability of expert guidance for specific countries is scarce [17, 18], especially in low- to middle-income countries.

In Malaysia, medicines risk communication has been carried out by the Pharmacovigilance Section of the National Pharmaceutical Regulatory Agency (NPRA) for over 30 years [19]. As the national regulatory authority, NPRA is tasked with ensuring the quality, efficacy and safety of pharmaceutical products in Malaysia. Currently, NPRA uses various risk communication methods including a national bulletin, safety alerts via email or social media, product package inserts, Direct Healthcare Professional Communication letters, press releases, and educational materials. Some of these communication materials are disseminated by pharmaceutical companies or other government agencies such as the Pharmacy Services Program, in collaboration with NPRA [19]. The majority of NPRA risk communication targets doctors and pharmacists, who perform the main roles of prescribing and dispensing medicines in Malaysia [20]. However, our previous analysis of real-world data revealed that the current risk communication efforts did not have a significant impact on ADR reporting and prescribing practice [21]. A nationwide survey conducted in 2021 also showed that awareness of risk communication methods among Malaysian doctors and pharmacists is generally low [19].

Factors that predict the usefulness of risk communication methods, preferences of respondents and suggestions to improve the risk communication have been previously published [19]. We have identified a list of strategies to enhance medicines risk communication in Malaysia through our previous survey [19] and systematic review [22]. However, a consensus from risk communication experts and recipients on which strategies should be prioritised in Malaysia has never been obtained. Faced with challenges such as budget constraints, lack of manpower, and lack of expertise [19, 23], it is impossible for regulators to simultaneously implement all the strategies identified. Therefore, our study aims to establish a consensus on the priority strategies to enhance risk communication about medicines by Malaysian regulators, using a modified Delphi survey with input from communicators and recipients.

## Methods

This study is registered under the National Institute of Health [NMRR ID-23-00478-BN0] and received ethical approval from the Medical Research and Ethics Committee, Ministry of Health Malaysia. We conducted and reported this Delphi study following the guidance provided in the Conducting and REporting DElphi Studies (CREDES) checklist (see Additional File 1: Supplementary Table S1) [24, 25].

### Study design

We conducted a two-round modified Delphi survey from March to June 2023, involving local and international panellists. The Delphi technique is a structured process that synthesises opinions of panellists to develop a group consensus [26, 27]. We used this technique to reduce bias because other group decision-making processes such as focus groups may be affected by the presence of individuals who dominate the discussion, or group pressure for compromise [28]. The Delphi technique addresses these limitations by using questionnaires to preserve anonymity of panellists, and providing controlled feedback in a multi-stage process which gives panellists the option to reassess their initial responses based on the aggregate group response [26, 29].

### Study participants

Our Delphi panel comprised various stakeholders of medicines risk communication, namely (i) communicators and (ii) recipients of risk communication related to medicines. We included both local and international communicators to ensure a range of expertise and diverse perspectives. It has been suggested that diversity in the demographic characteristics and professional experience of Delphi panellists could be beneficial [30–32]. This allows consensus to be based on multiple independent sources of opinion.

Under the category of “communicators”, we included senders of risk communication such as representatives from NPRA and international regulatory agencies, the Malaysian Pharmacy Services Programme, pharmaceutical industry representatives, public health consultants, and medicine communication experts. The communicators were required to have a minimum of 3 years’ experience in risk communication. We included experts from international bodies with vast experience communicating the risks of medicines, such as the World Health Organisation (WHO), EMA, the International Society of Pharmacovigilance (ISoP), and TGA Australia. Communication experts from the Health Sciences Authority (HSA) Singapore, which shares cultural similarities with Malaysia, were also surveyed.

Academicians and healthcare professionals (general practitioners, medical officers, clinical specialists, and pharmacists) from both the public and private sectors in Malaysia who have received and read NPRA risk communication were included under the category of “recipients”. We excluded those who were not comfortable answering in English, and recipients who were not aware of NPRA risk communication.

The target minimum sample size in this study was set at 30 respondents based on previous Delphi studies with similar aims and recommendations that suggest an optimal range of 20 to 60 panellists for health science-related Delphi studies [25, 29, 33, 34]. We used purposive sampling and snowballing to recruit members of the Delphi panel. Individual invitation emails containing a participant information sheet and briefing video were sent to 53 panellists in February or March 2023, before the start of Round 1.

Participation in this research was entirely voluntary. We provided all panellists with information on the study aims, procedures, risks, benefits, and protection for individual privacy. Informed consent was obtained from all participants at the beginning of the questionnaire.

### Questionnaire development

The traditional Delphi method involves idea gathering in Round 1 through open-ended questions [35]. However, we omitted this conventional first round of the study. Instead, we generated a list of strategies on enhancing risk communication through our previous research [19, 22, 36]. We identified an initial list of 43 strategies to enhance medicines risk communication in Malaysia through the findings of our survey [19], systematic review [22] and literature review. We performed face and content validity with a group of six experts on medicines risk communication from NPRA, the Ministry of Health Malaysia, and academia. The initial questionnaire containing 43 strategies was sent individually to the six experts, who were asked to provide qualitative feedback on each strategy. As an outcome of these validity tests, eight strategies were excluded because they could be merged under another strategy in the list. We also added two new strategies suggested by the experts. Thus, the list of strategies was refined to a total of 37 strategies [36], and categorised into six domains related to enhancing communication about the risks of medicines, as follows: (1) improve format and content; (2) implement educational programmes; (3) integrate risk communication information into practice; (4) increase use of technology; (5) evaluate effectiveness; and (6) increase collaboration (see Additional File 1: Supplementary Table S2 for details). We then pilot-tested the modified questionnaire among six members of the target population to finalise the Round 1 Delphi survey (see questionnaire in Additional File 2). The six members comprised two international communicators, one local communicator, and three recipients of Malaysian medicines risk communication. Feedback from the pilot test resulted in rewording several strategies and adding examples to enhance clarity. However, there was no change in the number of strategies included.

We designed and managed the questionnaire using Research Electronic Data Capture (REDCap) tools hosted at Universiti Malaya, Kuala Lumpur, Malaysia [37, 38]. REDCap is a secure, web-based software platform designed to support data capture for research studies, providing (1) an intuitive interface for validated data capture; (2) audit trails for tracking data manipulation and export procedures; (3) automated export procedures for seamless data downloads to common statistical packages; and (4) procedures for data integration and interoperability with external sources.

### Delphi round 1

In Round 1, we sent emails containing the link for the web-based survey to 53 individual panellists who had agreed to join the study. Panellists were shown the list of 37 strategies to enhance NPRA risk communication and asked to prioritise each strategy using a 5-point Likert scale (1- not a priority, 2- low priority, 3- medium priority, 4- high priority, 5- highest priority) [36]. We also provided open-ended columns in each domain for panellists to suggested additional strategies which were not in the list. Reminder emails were sent to non-responders at days 7 and 14 following the initial survey distribution.

To allow for near-consensus strategies to be reconsidered in Round 2, we set a cut-off mean priority score of ≥ 3.75 in the first round [39, 40]. All strategies which achieved this score were included in the Round 2 questionnaire, along with any additional strategies proposed by the panel.

### Delphi round 2

**Fig. 1:**
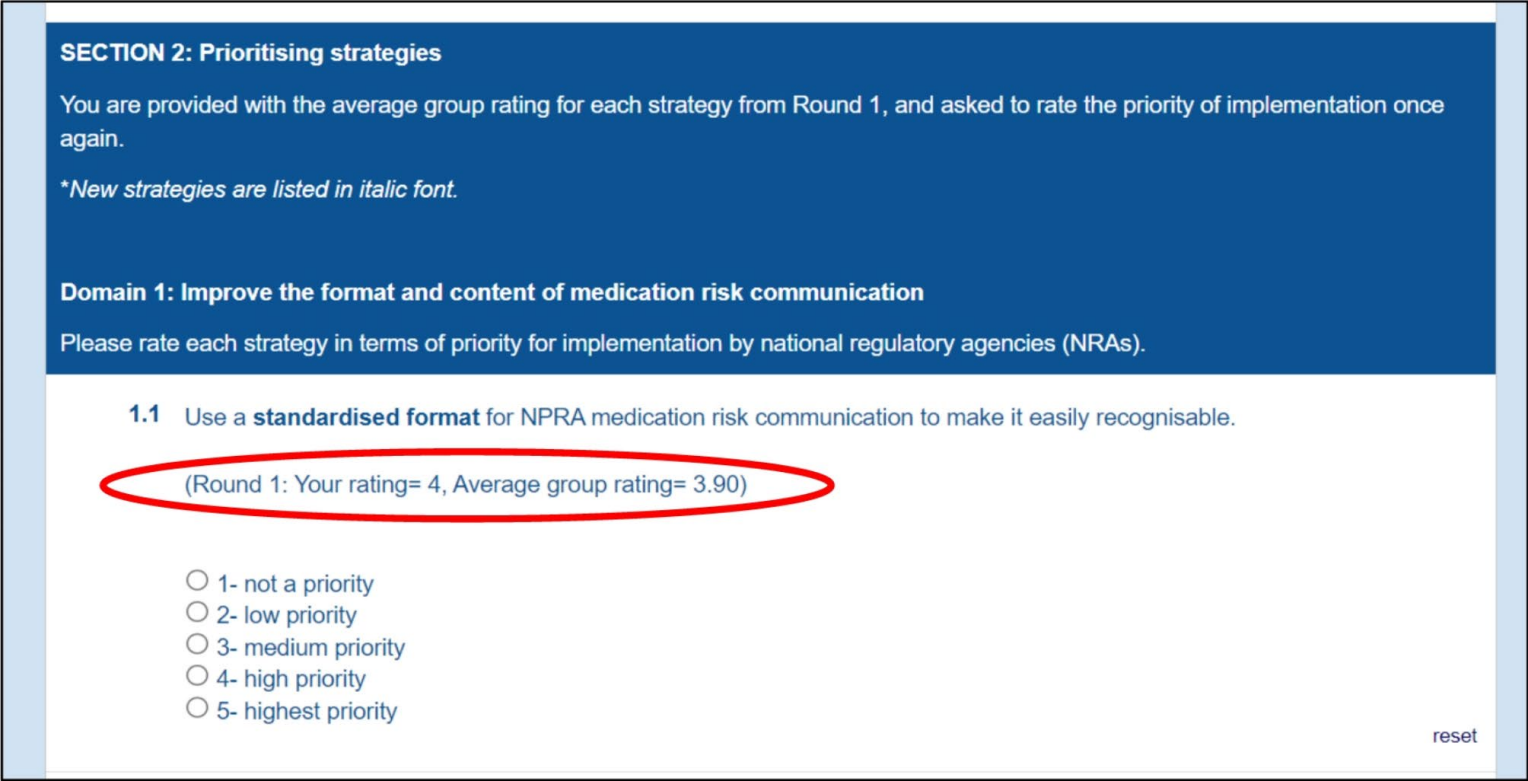
Sample of individualised feedback provided in the questionnaire for Round 2 of the Delphi survey. NPRA: National Pharmaceutical Regulatory Agency, NRA: national regulatory agency

Among the 53 invited panellists, only 42 completed the survey in Round 1. All 42 panellists who completed Round 1 were sent an email invitation to participate in the Round 2 survey. Our Round 2 questionnaire (see Additional File 3) contained a list of 23 initial strategies and 11 new strategies proposed in Round 1. For each of the initial strategies, panellists were given feedback on the mean group response as well as their personal response from Round 1 (Fig. 1: sample questionnaire Round 2) and asked to prioritise each strategy again using the same 5-point Likert scale. Two reminder emails were sent to non-responders and we received 39 responses. We determined the criteria for consensus a priori as ≥ 75% of panellists [27, 41] giving the strategy a rating of 4 (high priority) or 5 (highest priority) on the 5-point Likert scale [36].

### Data analysis

All data obtained was analysed using IBM SPSS version 20. We used descriptive statistics to present the demographic characteristics of panellists. We conducted thematic analysis to identify and categorise additional strategies suggested in Round 1 through open-ended columns.

From Round 2, we calculated the mean score of each strategy, and percentage of panellists who rated each strategy as 4 (high priority) or 5 (highest priority) on the 5-point Likert scale. We also assessed each strategy for disagreement among the panellists in both rounds. Disagreement is defined as ≥ 30% of participants rating a strategy 1 or 2 on the Likert scale, and ≥ 30% rating it 4 or 5 [42, 43].

We compared the standard deviations of priority scores for each strategy in both rounds to determine the extent of agreement. To assess the shift in opinion by the panellists between Round 1 and Round 2, we calculated the median degree of opinion change [44].

The data was also analysed separately according to subgroups. We used the Pearson Chi-squared test of independence to compare the priority rating between two subgroups: (i) communicators versus recipients, and (ii) local versus international panellists. Level of significance (α) was set at 0.05. Scatter plots were used to compare the difference of strategy prioritisation between the subgroups.

## Results

**Fig. 2:**
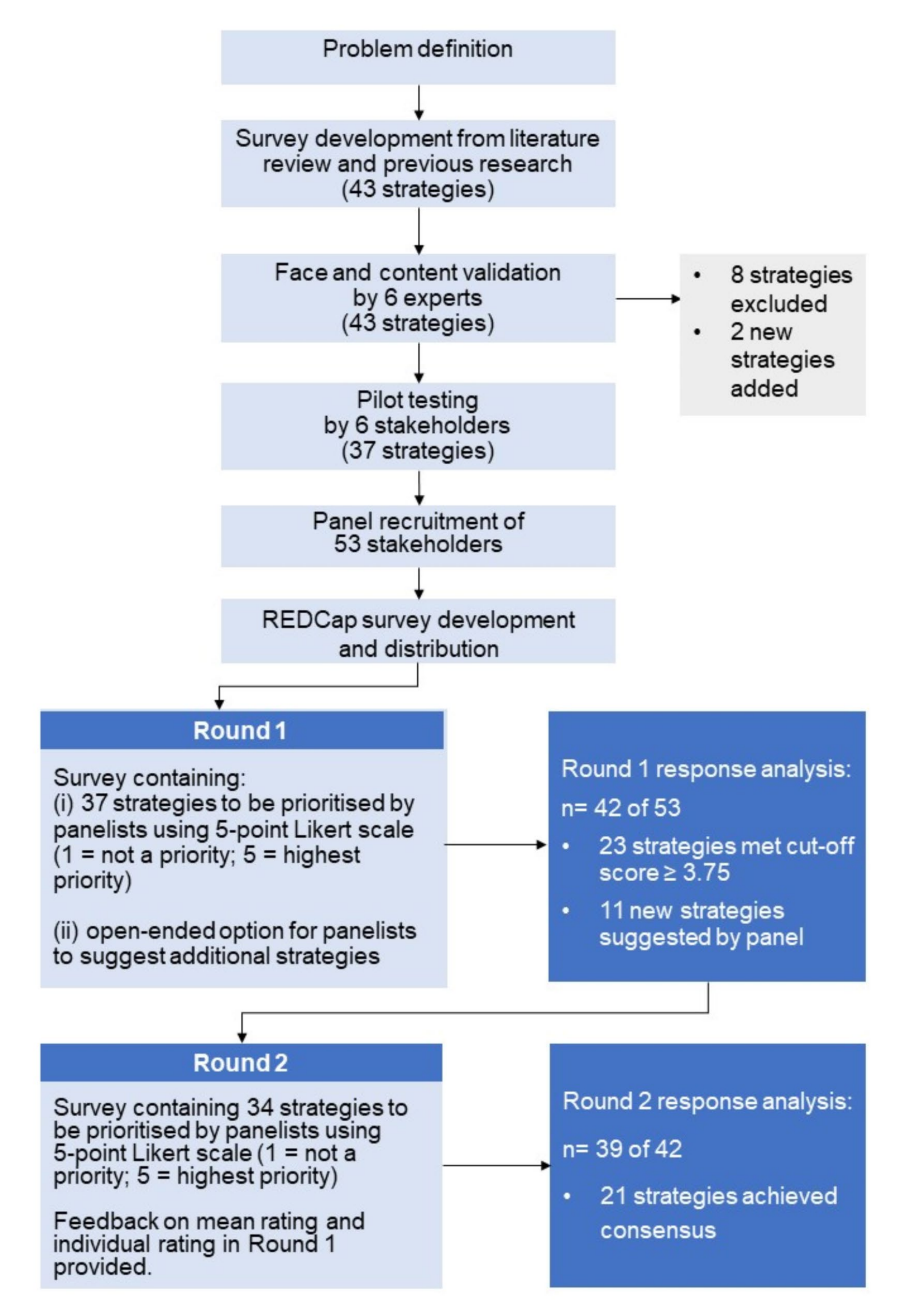
Flowchart of modified Delphi study to prioritise strategies on enhancing medicines risk communication. REDCap: Research Electronic Data Capture tool

Figure 2 illustrates the modified Delphi process employed in this study. A total of 48 strategies were assessed by our panellists (37 strategies identified through literature review and our previous research; 11 additional strategies from the panel in Round 1).

Table 1 shows the socio-demographic characteristics and response rates of our Delphi survey participants. A total of 42 stakeholders from nine countries participated in Round 1 of this survey, comprising 27 communicators (64.3%) and 15 recipients of medicines risk communication (35.7%). The average response rate for Round 1 was 79.2% (communicators 84.3%, recipients 71.4%) and 92.9% in Round 2 (communicators 96.3%, recipients 86.7%). Detailed characteristics of the panellists are listed in Additional File 1: Supplementary Table S3. More than 85% of our respondents had above 10 years work experience in their fields of expertise.

**Table 1:** Response rates and socio-demographics

Demographics	Communicators		Recipients	
	Round 1 (*n* = 27) *n* (%)	Round 2 (*n* = 26) *n* (%)	Round 1 (*n* = 15) *n* (%)	Round 2 (*n* = 13) *n* (%)
**Participant response rate**	27/32 (84.3)	26/27 (96.3)	15/21 (71.4)	13/15 (86.7)
**Gender**				
Female	24 (88.9)	23 (88.5)	8 (53.3)	7 (53.8)
Male	3 (11.1)	3 (11.5)	7 (46.7)	6 (46.2)
**Age (years)**				
Mean ± SD	44.7 ± 6.3	44.9 ± 6.3	48.1 ± 10.4	48.1 ± 11.0
31–40	9 (33.3)	8 (30.8)	3 (20.0)	3 (23.1)
41–50	13 (48.2)	13 (50.0)	7 (46.7)	6 (46.2)
51–60	5 (18.5)	5 (19.2)	2 (13.3)	1 (7.6)
61–70	0 (0)	0 (0)	3 (20.0)	3 (23.1)
**Designation**				
Communicator	8 (29.6)	7 (26.9)	0 (0)	0 (0)
Doctor	5 (18.5)	5 (19.2)	7 (46.7)	5 (38.5)
Pharmacist	14 (51.9)	14 (53.9)	8 (53.3)	8 (61.5)
**Location of work**				
Malaysia	11 (40.8)	10 (38.5)	15 (100)	13 (100)
Global (based in Malaysia, Netherlands, Oman, Philippines and Switzerland)	7 (25.9)	7 (26.9)	0 (0)	0 (0)
Australia	3 (11.1)	3 (11.5)	0 (0)	0 (0)
Singapore	3 (11.1)	3 (11.5)	0 (0)	0 (0)
Sweden	2 (7.4)	2 (7.7)	0 (0)	0 (0)
Italy	1 (3.7)	1 (3.9)	0 (0)	0 (0)
**Experience in field of expertise (years)**				
3–5	1 (3.7)	1 (3.8)	0 (0)	0 (0)
6–10	4 (14.8)	3 (11.5)	1 (6.6)	1 (7.6)
11–20	16 (59.3)	16 (61.5)	7 (46.7)	6 (46.2)
Above 20	6 (22.2)	6 (23.2)	7 (46.7)	6 (46.2)

SD: standard deviation

### Selection of priority strategies

Following Round 1, 23 strategies with a mean priority score ≥ 3.75 (as shown in Additional File 1: Supplementary Table S2) were included in Round 2, along with 11 new strategies suggested by the panel (indicated with asterisks in Additional File 1: Supplementary Table S4) [36]. In Round 2, 21 out of the 34 strategies evaluated (61.8%) achieved consensus with ≥ 75% of panellists rating each strategy as 4 (high priority) or 5 (highest priority).

**Fig. 3:**
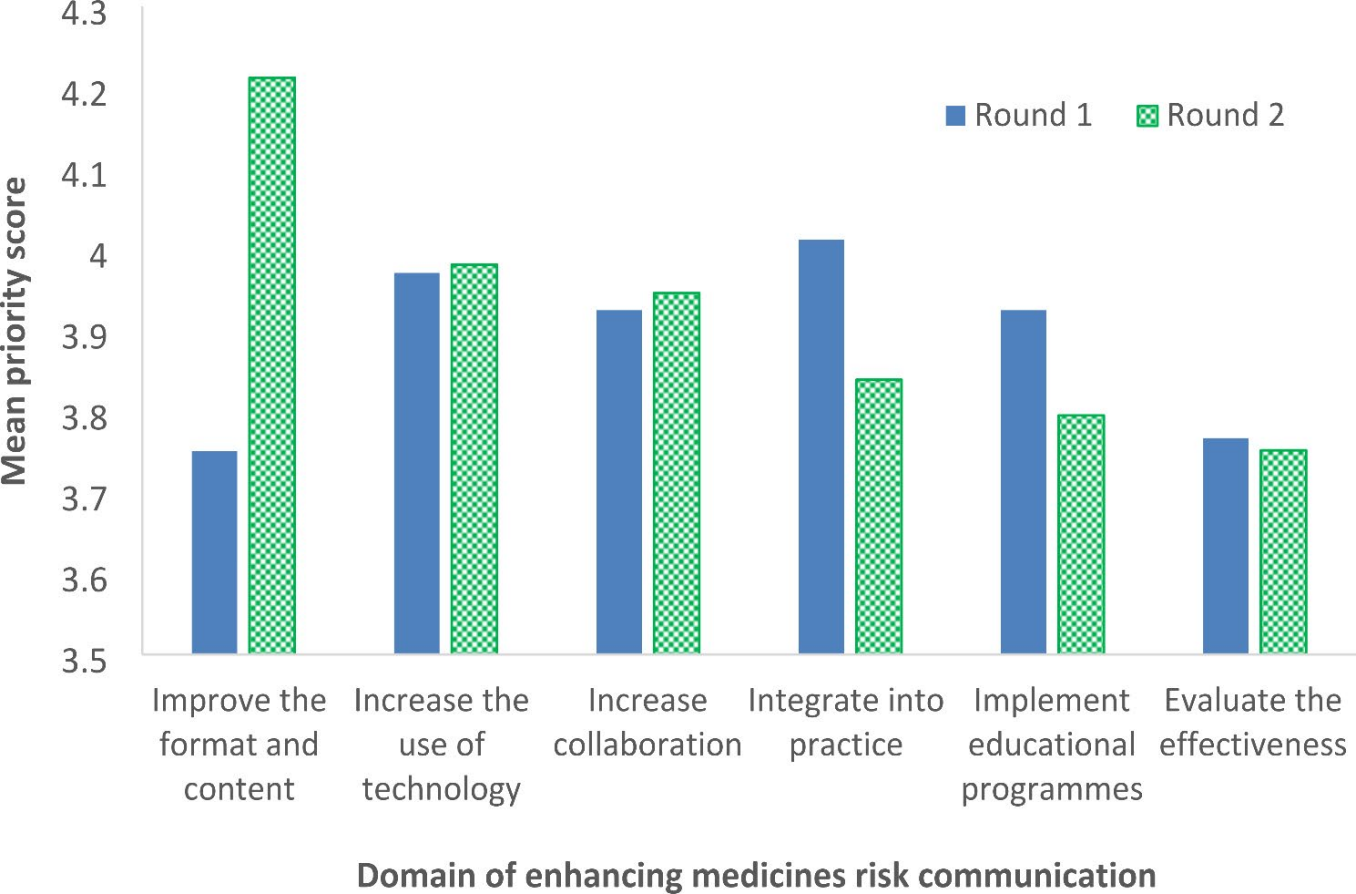
Mean priority scores for each domain of enhancing medicines risk communication in both rounds of the Delphi survey

Figure 3 shows the mean priority scores for each domain in Round 1 and Round 2. Following Round 2, the top three domains were to (1) improve the format and content of risk communication, (2) increase the use of technology, and (3) increase collaboration to enhance risk communication about medicines. In Round 1, the domain “improve format and content” of risk communication contained nine strategies and ranked as the lowest priority domain. This domain emerged as top priority in Round 2, containing four strategies, and showed the greatest increase in mean priority score between the two rounds. The lower ranked strategies in Round 1 included to “establish an external medicines risk communication advisory board to review regulatory risk communication” and “increase use of narrative-style messages”. Many panellists commented that existing advisory boards on medicines safety may be able to review risk communication, but it may not be feasible to obtain the approval of an advisory board for each publication.

**Fig. 4:**
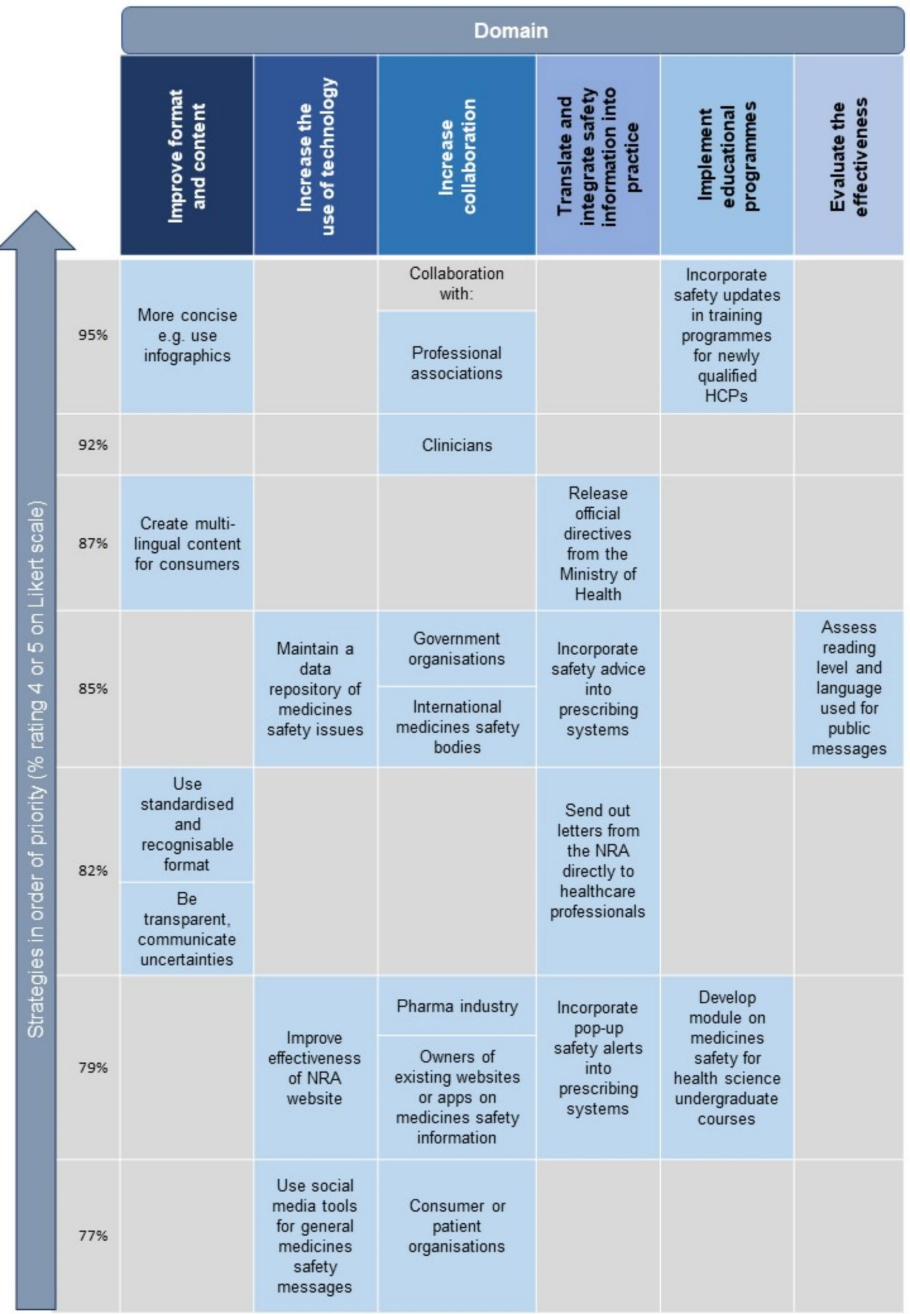
Priority strategies for enhancing risk communication about medicines as identified through this Delphi study. HCP: healthcare professional, NRA: national regulatory agency

Figure 4 provides a summary of the list of strategies for enhancing risk communication about medicines in Malaysia as identified through our Delphi study, in order of priority for each domain. Please refer to Additional File 1: Supplementary Table S5 for the full list of priority strategies. Further details of the mean scores, percentage ratings and subgroup comparisons for each strategy are shown in Additional File 1: Supplementary Table S6.

### Extent of agreement

Our analysis identified one strategy on which the panellists showed disagreement, namely the strategy “to offer incentives to pharmaceutical companies which maintain effective systems for medicines risk communication”. This strategy was rated 1 or 2 on the Likert scale by 33% of panellists, and rated 4 or 5 by another 33% of panellists (see Additional File 1: Supplementary Table S6).

We found that the standard deviations decreased from the first to second round for 21 out of the 23 strategies which were included in both rounds of this Delphi survey (see Additional File 1: Supplementary Table S6). As shown in Table 2, there was a minimal shift in opinion by the panellists between Round 1 and Round 2. The median degree of opinion change for most panellists was 0. None of the panellists had a median opinion change of more than 1. We found no significant difference between communicators and recipients with regards to the median degree of opinion change [ χ^2^_(1, *N*=39)_ = 0.315; *p* = 0.575].

**Table 2:** Degree of opinion change from round 1 to round 2 of the Delphi study

Category	*n* with median degree of opinion change				
	0	1	2	3	4
Communicators (*n* = 26)	20	6	0	0	0
Recipients (*n* = 13)	11	2	0	0	0

### Subgroup analysis

**Fig. 5:**
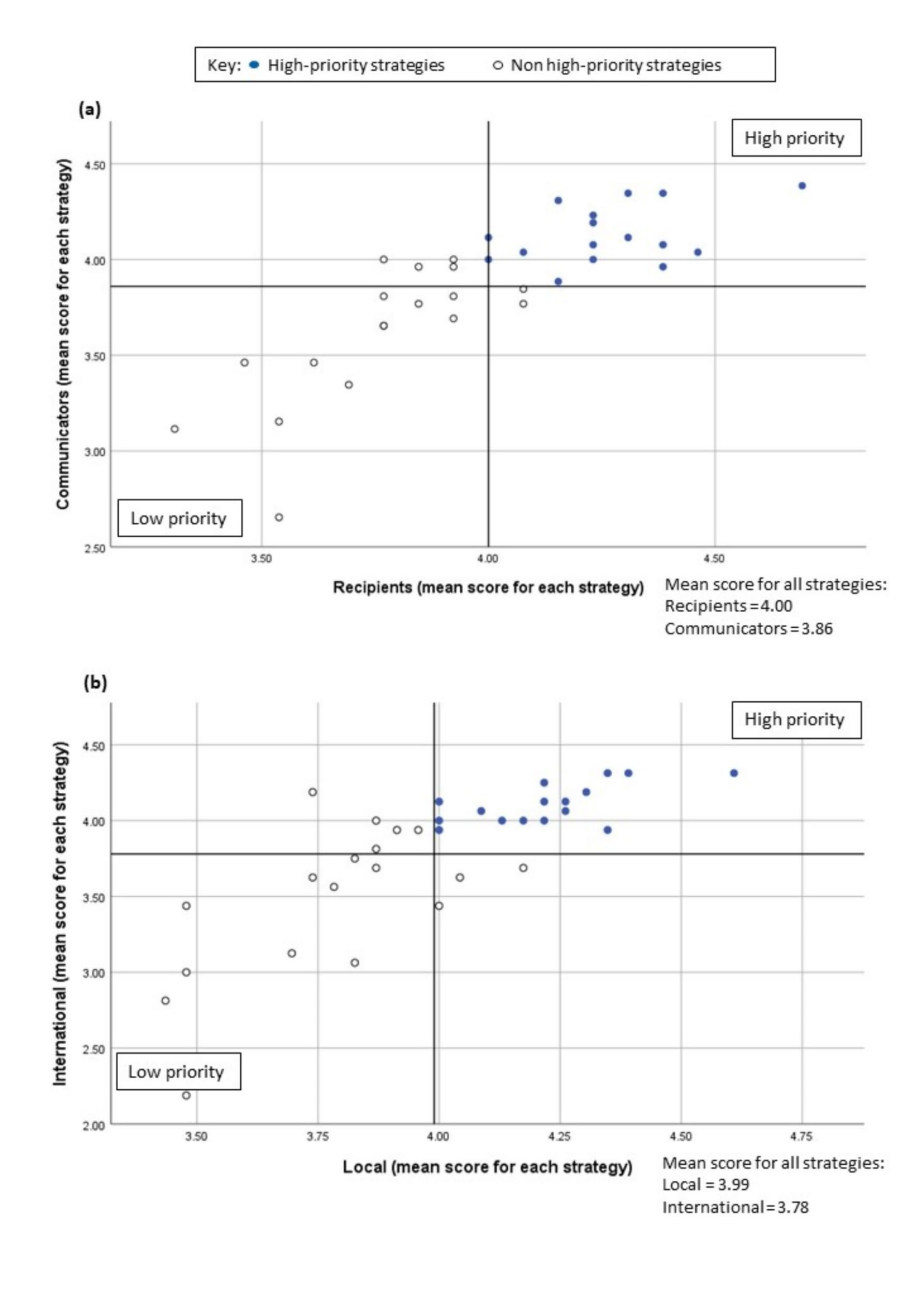
Comparison of the mean priority score given by respondents for each strategy in Round 2 of the Delphi study for: **(a)** Communicators versus recipients, and **(b)** Local (Malaysian) versus international respondents. The lines represent the mean scores of each subgroup for all strategies in Round 2

Figure 5 illustrates our subgroup analysis comparing the mean priority scores for each strategy in Round 2 between (i) communicators versus recipients [Fig. 5(a)]; and (ii) local versus international panellists [Fig. 5(b)] (see Additional File 1: Supplementary Table S7 for details). We found that 16 strategies fell within the high priority area for each comparison group, as indicated by the blue dots in Fig. 5. All these strategies were among the 21 strategies which achieved consensus through our Delphi process (Fig. 4).

There was a statistically significant difference in rating between subgroups for two strategies. First, local panellists gave significantly higher ratings compared to international panellists for the strategy “to increase outreach of educational programmes” [χ^2^_(1, *N* = 39)_ = 6.4; *p* = 0.040] [Fig. 5(b)].

Second, priority ratings for the strategy “to offer incentives to pharmaceutical companies which maintain effective systems for medicines risk communication” were significantly higher among recipients compared to communicators [χ^2^_(1, *N*= 39)_ = 10.1; *p* = 0.039] [Fig. 5(a)] and among local versus international panellists [χ^2^_(1, *N*= 39)_ = 14.3; *p* = 0.007] [Fig. 5(b)].

## Discussion

Our Delphi study successfully established a consensus on the priority strategies to enhance risk communication about medicines by Malaysian regulators. We obtained a list of 21 priority strategies based on the opinions of medicines risk communication experts and recipients. The domains which obtained the highest mean scores, thus rated as domains to be prioritised are to “improve the format and content of risk communication”, “increase the use of technology”, and “increase collaboration to enhance risk communication about medicines”. Meanwhile, the top three highest rated strategies were to “create more concise communication”, “increase collaboration with professional associations”, and “incorporate medicines safety information in training programmes for newly qualified healthcare professionals”.

The strategy to produce concise communication with increased use of infographics fits with the current preferences of audiences for fast and succinct information [45]. Previous studies have shown that the use of visuals helps recipients understand complex information more quickly compared to text alone [46]. This is supported by several theories including the cognitive theory of multimedia learning [47], and the dual coding theory [48]. The design of effective infographics should involve highlighting essential information, reducing extraneous information, using space effectively, and inserting appealing graphics [47, 49].

Collaboration is essential to produce an effective system for communicating the risks of medicines. In Round 2 of this Delphi study, we found that seven of the eight strategies related to collaboration achieved consensus to be prioritised. These findings highlight the importance of collaboration between regulators and various stakeholders to improve content and widen dissemination of the risk communication. Previous studies and guidelines have established that collaboration between regulators, healthcare professionals, patients, the pharmaceutical industry and policy makers is needed to increase communication effectiveness [1, 14].

Panellists in this Delphi study also prioritised the incorporation of medicines safety information in training programmes for newly qualified healthcare professionals. The integration of such information should be made mandatory in orientation programmes and repeated at regular intervals to ensure all healthcare professionals are aware of the latest sources of medicines safety information [50]. Another high-priority strategy was to develop a module on medicines safety for undergraduate health science courses. A team of experts in medicines safety from the WHO and ISoP published a curriculum for teaching pharmacovigilance in 2014 [51]. However, this curriculum needs to be revised to reflect the evolvement of pharmacovigilance over the past decade in terms of scientific, regulatory and technological developments [51]. Additionally, globalization has led to the increased availability of effective but potentially harmful medicinal products across various countries. This situation requires that healthcare workers from multiple disciplines have updated knowledge of pharmacovigilance. Thus, pharmacovigilance should be included as a standard module in health science courses worldwide [51].

Among the 48 strategies assessed by our Delphi panel, 27 strategies were dropped. The domain “improve format and content” showed the largest increase in mean score from Round 1 to Round 2, after six lower ranked strategies were excluded following the first round. All four strategies included in this domain for Round 2 emerged as high-priority, indicating the importance of creating concise, multi-lingual communication using a standardised format, and being transparent in communicating uncertainties. However, some interesting strategies were not rated as high priority in our Delphi study. For example, the strategies to “leverage artificial intelligence tools to make searching easier and generate more interactive communication” and “use a mobile phone application for medicines risk communication” did not meet the criteria to be prioritised. This was surprising given the rapid development in generative artificial intelligence that has made the use of chatbots and machine learning more mainstream [52]. Based on the results of our study, the current focus should be on improving the format of risk communication, enhancing the regulatory agency website, improving training on pharmacovigilance particularly for doctors and pharmacists, and increasing collaboration with various stakeholders. Globally, we observe an increase in the use of artificial intelligence to communicate the risks associated with medicines [53]. However, our Delphi findings indicate that Malaysian regulators should prioritise the basic requirements of effective risk communication, such as format, outreach and training.

Our Delphi study revealed differing opinions among subgroups. For instance, regarding the strategy “to offer incentives to pharmaceutical companies which maintain effective systems for medicines risk communication”, there were notable differences. Recipients rated this suggestion as higher priority compared to communicators, and local panellists rated it higher than international panellists. Additionally, among all strategies, the strategy to offer incentives was the only strategy where there was significant disagreement among panellists. Many regulators and international panellists rated this strategy as low priority. We postulate that these panellists believe that pharmaceutical companies need to strike a balance between expecting incentives, and performing their duty to maintain competent risk communication systems [54]. Local panellists also gave higher ratings than international panellists for the strategy “to increase outreach of educational programmes (for example to private sector healthcare professionals)”. This may be because local panellists were more aware of the scenario in Malaysia, where until recently, educational programmes organised by the public sector did not routinely involve participants from the private sector. These differences in opinion between panellists emphasise the need for a standardised list of priority strategies developed by experts and multiple stakeholders.

Although our panellists were asked to consider the strategies in the context of Malaysian regulatory risk communication, the list of priority strategies also potentially serves as a useful guide for other countries aiming to improve their risk communication about medicines. Almost all high-income countries have robust pharmacovigilance systems in terms of monitoring adverse drug reactions [13]. However, establishing effective systems for risk communication about medicines remains a challenge for most countries [7, 11], particularly due to budget constraints in low- or middle-income countries [13]. Individual countries need to develop specific strategies which help regulators, manufacturers and healthcare professionals overcome the challenges of risk communication, including misinformation and public mistrust [55].

This study adds a suite of implementable strategies produced by a group of medicines risk communication experts and stakeholders. The impact of this study will be seen in terms of increased patient safety and cost-savings. Following this study, clear steps could be taken to improve the quality of risk communication and educational material in Malaysia, ensuring both healthcare professionals and patients are as well-informed as possible. The aim is to go beyond diffusion of information, creating a two-way process allowing patients to use the information effectively and promote empowerment [1]. Implementation of each strategy must be followed with evaluation of its impact, for example on prescribing behaviour, knowledge, or ADR reporting rate. This evaluation will provide valuable evidence to amend and improve the strategies [1].

### Strengths and limitations

We used the modified Delphi technique to reduce bias due to dominant personalities, and allow consensus building between experts from various geographical locations or time zones. To ensure we considered all relevant strategies, panellists were allowed to suggest additional strategies in Round 1 via free-text comments. There was a low median degree of change in opinion, indicating stability as the majority of panellists did not change their opinion between the two rounds. Another strength of our study is the inclusion of communication experts from nine countries as well as recipients who are familiar with the current Malaysian regulatory risk communication. Our response rates ranged from 71 to 96%, which further strengthens the study.

Our study has several limitations. First, we presented a list of strategies to the panel rather than conducting the traditional open-ended first round. This was done to ensure the strategies were relevant to the Malaysian regulatory context but could potentially have stifled the generation of ideas by panellists. To overcome this limitation, panellists were invited to suggest additional strategies in Round 1. However, we found that most new suggestions were not given a high priority rating. Only two new strategies achieved consensus, namely collaboration with international bodies, and communicating transparently. This indicates that the initial list of strategies could be considered comprehensive. Second, priority strategies were identified but not ranked by the panellists. Considering the varied backgrounds of our panellists, we did not require the strategies to be ranked as this would require in-depth knowledge of the current Malaysian risk communication. The scope of this paper also does not encompass detailed guidance on how these strategies could be implemented. Participants were not asked to consider the feasibility of implementation, or consider barriers such as cost and manpower. While we looked at strategies to enhance risk communication by Malaysian regulators, some of the strategies fall under the jurisdiction of other authorities, such as policy makers and academicians. Our study aimed to obtain the broad perspective of stakeholders on priority strategies. Following this Delphi survey, we will conduct an expert panel discussion to develop a strategic plan for enhancing risk communication about medicines in Malaysia. Third, as medicines risk communication is an ever-developing field, the strategies identified are deemed to be of highest priority at the time the study was undertaken. This list of strategies needs to be reviewed and updated periodically, for example every five years. Fourth, while our study focused on doctors and pharmacists as they are currently the main recipients of Malaysian medicines risk communication, input from other stakeholders such as nurses, dentists, and consumers may have added to the range of strategies. Further studies are being planned to evalute the implementation of strategies and involve other stakeholders. Fifth, our Delphi panel was restricted to those comfortable answering in the English language, which may limit the generalisability of our study findings [56]. Nevertheless, our study offers an important list of priority strategies produced by experts, as a guide for enhancing medicines risk communication in Malaysia and other countries with growing pharmacovigilance systems.

### Future research

Future studies should involve stakeholders other than doctors and pharmacists, especially nurses and consumers. We need to design studies to evaluate each of the strategies implemented, for example via feedback surveys or the analysis of adverse drug reaction databases. Further study is also required on the barriers to implementation of strategies. Collaborative research should be conducted with regulatory authorities from other countries with similar cultures or economic status, to accelerate the progress of risk communication systems.

## Conclusions

Combining the views of Malaysian and international medicines communication experts and also recipients of Malaysian regulatory risk communication, our Delphi study produced a consensus-based list of priority strategies for enhancing risk communication about medicines in Malaysia. This list was used to develop a strategic plan for implementation by the Malaysian regulatory authority, which would also be potentially adaptable to other countries with developing pharmacovigilance systems. The strategies identified as of top priority were to create more concise communication, increase collaboration with professional associations, and incorporate medicines safety information in training programmes for newly qualified healthcare professionals. The existence of different views between communicators and recipients, and also between local and international panellists, highlights the importance of involving multiple stakeholders in research.

## Electronic supplementary material

Below is the link to the electronic supplementary material.

Supplementary Material 1. Additional file 1: Supplementary information

Supplementary Material 2. Additional file 2: Round 1 Delphi questionnaire

Supplementary Material 3. Additional file 3: Round 2 Delphi questionnaire

## Data Availability

All data generated or analysed during this study are included in this published article and its electronic supplementary material.
