# Supplementary material for: Strategies to enhance risk communication about medicines in Malaysia: a Delphi study among multinational experts
Source: BMC Health Serv Res. 2024 Sep 3;24:1019. doi: 10.1186/s12913-024-11476-0 (PMC11373486; doi:10.1186/s12913-024-11476-0)
Supplement: Supplementary file 1 — Supplementary Material 1. Additional file 1: Supplementary information [file 12913_2024_11476_MOESM1_ESM.docx]

**Strategies to enhance risk communication about medicines in Malaysia: A Delphi study among multinational experts**

**Additional file 1: Supplementary material**

**Content:**

**Supplementary Table S1** - Conducting and REporting DElphi Studies (CREDES) checklist

**Supplementary Table S2 -** List of strategies and mean priority scores for Round 1 of the Delphi study

**Supplementary Table S3 -** Characteristics of Delphi panelists

**Supplementary Table S4 -** List of strategies for Round 2 of the Delphi study

**Supplementary Table S5 -** List of priority domains and strategies following Round 2 of the Delphi survey

**Supplementary Table S6 -** Data analysis (Round 1 and 2 of the Delphi study)

**Supplementary Table S7 -** Subgroup analysis comparing the mean priority scores for each strategy in Round 2 between (i) communicators versus recipients and (ii) local versus international panellists

**Supplementary Table S1** Conducting and REporting DElphi Studies (CREDES) checklist

| **Theme and Recommendations** | | **Checklist** | **Reported in manuscript** |
| --- | --- | --- | --- |
| Rationale for the choice of the Delphi technique | | | |
|  | Justification. The choice of the Delphi technique as a method of systematically collating expert consultation and building consensus needs to be well justified. When selecting the method to answer a particular research question, it is important to keep in mind its constructivist nature. | 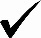 | Section 2.1 |
| Planning and design | | | |
|  | Planning and process. The Delphi technique is a flexible method and can be adjusted to the respective research aims and purposes. Any modifications should be justified by a rationale and be applied systematically and rigorously. | 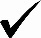 | Section 2  Figure 2 |
|  | Definition of consensus. Unless not reasonable due to the explorative nature of the study, an a priori criterion for consensus should be defined. This includes a clear and transparent guide for action on (a) how to proceed with certain items or topics in the next survey round, (b) the required threshold to terminate the Delphi process and (c) procedures to be followed when consensus is (not) reached after one or more iterations. | 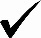 | Section 2.4 and 2.5 |
| Study conduct | | | |
|  | Informational input. All material provided to the expert panel at the outset of the project and throughout the Delphi process should be carefully reviewed and piloted in advance in order to examine the effect on experts’ judgements and to prevent bias. | 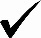 | Section 2.2 |
|  | Prevention of bias. Researchers need to take measures to avoid directly or indirectly influencing the experts’ judgements. If one or more members of the research team have a conflict of interest, entrusting an independent researcher with the main coordination of the Delphi study is advisable. | 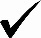 | Section 2.1 |
|  | Interpretation and processing of results. Consensus does not necessarily imply the ‘correct’ answer or judgement; (non)consensus and stable disagreement provide informative insights and highlight differences in perspectives concerning the topic in question. | 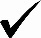 | Section 3.1  Section 4 |
|  | External validation. It is recommended to have the final draft of the resulting guidance reviewed and approved by an external board or authority before publication and dissemination. |  | In process of review by NPRA Malaysia |
| Reporting | | | |
|  | Purpose and rationale. The purpose of the study should be clearly defined and demonstrate the appropriateness of the use of the Delphi technique as a method to achieve the research aim. A rationale for the choice of the Delphi technique as the most suitable method needs to be provided. | 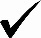 | Section 1  Section 2.1 |
|  | Expert panel. Criteria for the selection of experts and transparent information on recruitment of the expert panel, sociodemographic details including information on expertise regarding the topic in question, (non)response and response rates over the ongoing iterations should be reported. | 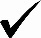 | Section 2.2  Table 1  Online resource 3 |
|  | Description of the methods. The methods employed need to be comprehensible; this includes information on preparatory steps (How was available evidence on the topic in question synthesised?), piloting of material and survey instruments, design of the survey instrument(s), the number and design of survey rounds, methods of data analysis, processing and synthesis of experts’ responses to inform the subsequent survey round and methodological decisions taken by the research team throughout the process. | 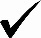 | Section 2 |
|  | Procedure. Flow chart to illustrate the stages of the Delphi process, including a preparatory phase, the actual ‘Delphi rounds’, interim steps of data processing and analysis, and concluding steps. | 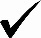 | Figure 2  Section 2 |
|  | Definition and attainment of consensus. It needs to be comprehensible to the reader how consensus was achieved throughout the process, including strategies to deal with non-consensus. |  |  |
|  | Results. Reporting of results for each round separately is highly advisable in order to make the evolving of consensus over the rounds transparent. This includes figures showing the average group response, changes between rounds, as well as any modifications of the survey instrument such as deletion, addition or modification of survey items based on previous rounds. | 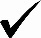 | Table 1  Section 3.1  Online resources 2, 4, 5, 6 |
|  | Discussion of limitations. Reporting should include a critical reflection of potential limitations and their impact of the resulting guidance. | 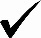 | Section 4.1 |
|  | Adequacy of conclusions. The conclusions should adequately reflect the outcomes of the Delphi study with a view to the scope and applicability of the resulting practice guidance. | 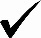 | Section 5 |
|  | Publication and dissemination. The resulting guidance should be clearly identifiable from the publication, including recommendations for transfer into practice and implementation. If the publication does not allow for a detailed presentation of either the resulting practice guidance or the methodological features of the applied Delphi technique, or both, reference to a more detailed presentation elsewhere should be made (e.g. availability of the full guideline from the authors or online; publication of a separate paper reporting on methodological details and particularities of the process (e.g. persistent disagreement and controversy on certain issues)). A dissemination plan should include endorsement of the guidance by professional associations and health care authorities to facilitate implementation. | 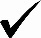 | Figure 4  Full strategic plan is in process of review by NPRA Malaysia |

**Supplementary Table S2** List of strategies and mean priority scores for Round 1 of the Delphi study

|  | **Strategy** | **Mean priority score** |
| --- | --- | --- |
| **Domain 1: Communication essentials- improve the format and content of medicines risk communication** | |  |
| 1.1 | Use a standardised format for medicines risk communication to make it easily recognisable. | 3.91 |
| 1.2 | More concise communication, e.g. increased use of infographics with links to full details. | 4.38 |
| 1.3 | Increase use of narrative-style messages e.g. storytelling to explain a case study | 3.36 |
| 1.4 | Increase original content e.g. local data or case studies | 3.74 |
| 1.5 | Make the communication more engaging, e.g. 1 minute videos with links to full details. | 3.67 |
| 1.6 | Create templates of risk communication messages for different situations (e.g. traffic light colour coding based on level of risk) | 3.57 |
| 1.7 | Hire staff or external experts with required expertise (e.g. communication experts, graphic designers, social media managers) | 3.67 |
| 1.8 | Create multi-lingual communication for consumers (e.g. English and Malay language). | 4.33 |
| 1.9 | Establish an external medicines risk communication advisory board to review NPRA risk communication. | 3.14 |
| **Domain 2: Implement educational programmes to increase awareness on medicines safety and regulatory risk communication** | |  |
| 2.1 | Conduct **regular** continuing medical education (CME) sessions for healthcare professionals. | 3.91 |
| 2.2 | Conduct **online** CME sessions for healthcare professionals. | 3.74 |
| 2.3 | Increase **outreach** of educational programmes or CME (e.g. to private sector healthcare professionals). | 3.88 |
| 2.4 | Develop online videos on medicines safety for **self-guided learning** by healthcare professionals. | 3.74 |
| 2.5 | Develop online videos on medicines safety to increase awareness among **consumers**. | 3.88 |
| 2.6 | Incorporate medicines safety information in training programmes for **newly qualified** healthcare professionals (e.g. medical house officers, provisionally-registered pharmacists). | 4.26 |
| 2.7 | Develop module on medicines safety for **undergraduates** (health science courses) | 4.07 |
| **Domain 3: Translate and integrate risk communication information into practice** | |  |
| 3.1 | Release **official directives** from the Ministry of Health to enforce risk communication about important medicines safety changes. | 4.12 |
| 3.2 | Send out **letters from the NRA** directly to healthcare professionals to reinforce communication about high risk medicines safety issues. | 3.79 |
| 3.3 | Incorporate **pop-up safety alerts** into electronic prescribing systems (e.g. new interactions or contraindications). | 4.02 |
| 3.4 | Incorporate **medicines safety recommendations** into electronic prescribing systems (e.g. counselling points). | 4.12 |
| **Domain 4: Increase the use of technology in medicines risk communication** | |  |
| 4.1 | Improve effectiveness of NRA website (e.g. create user-friendly interface, feedback form for risk communication) | 4.41 |
| 4.2 | Allow users to specify which area of interest they would like to receive medicines risk communication emails. | 3.64 |
| 4.3 | Establish and maintain **social media** tools for communicating general medicines safety information (especially with the public). | 4.07 |
| 4.4 | Use a mobile phone application for medicines risk communication (e.g. allow users to receive targeted or personalised updates) | 3.55 |
| 4.5 | Maintain a data repository of medicines safety issues (e.g. make it searchable, allow public access, include practice recommendations) | 4.19 |
| **Domain 5: Evaluate the effectiveness of medicines risk communication** | |  |
| 5.1 | Review detailed breakdown of NRA website usage statistics. | 3.55 |
| 5.2 | Assess the effectiveness of social media tools in reaching target audiences. | 3.83 |
| 5.3 | Assess the reading level and language used in public communication to ensure it is appropriate for the target audience. | 4.07 |
| 5.4 | Develop and conduct collaborative research on the impact of risk communication on healthcare professionals (e.g. changes in prescribing practice, ADR reporting rates, knowledge and awareness). | 3.74 |
| 5.5 | Develop and conduct collaborative research on the impact of risk communication on **consumers** (e.g. awareness, knowledge and reported behaviours). | 3.64 |
| **Domain 6: Increase collaboration to improve content and widen dissemination of medicines risk communication.**  Consider the collaboration of an NRA with the following stakeholders. | |  |
| 6.1 | Government organisations (e.g. Ministry of Health state directors, directors of hospitals and heads of departments assist in disseminating NRA communication to their staff) | 3.95 |
| 6.2 | Clinicians (e.g. practising doctors, pharmacists, dentists and nurses contribute articles on medicines safety or collaborate with NRA in research) | 4.29 |
| 6.3 | Academicians (e.g. collaborate in research, contribute articles and disseminate NRA communication) | 3.48 |
| 6.4 | Pharmaceutical industry (e.g. collaborate in research, contribute articles and disseminate NRA communication) | 3.81 |
| 6.5 | Professional associations (e.g. Malaysian Medical Association, Malaysian Pharmacists Society, Malaysian Dental Association, Malaysian Nurses Association: contribute articles and disseminate NRA communication) | 4.10 |
| 6.6 | Consumer or patient organisations (e.g. contribute articles and disseminate NRA communication) | 3.88 |
| 6.7 | Owners of existing websites or apps regularly used by consumers or healthcare professionals to obtain medicines safety information (e.g. link important NPRA safety information directly through existing websites such as MiMS) | 3.98 |

CME: continuing medical education; NPRA: National Pharmaceutical Regulatory Agency; NRA: national regulatory agency

**Supplementary Table S3** Characteristics of Delphi panelists

| **Panelist Code** | **Gender** | **Work experience (years)** | **Main place of work** | **Institution/ Setting** | **Designation** |
| --- | --- | --- | --- | --- | --- |
| P1 | F | 6 to 10 | Malaysia | Regulatory | Pharmacist |
| P2 | F | 11 to 20 | Malaysia | Regulatory | Pharmacist |
| P3 | F | 11 to 20 | Malaysia | Regulatory | Pharmacist |
| P4 | F | 6 to 10 | Malaysia | Regulatory | Pharmacist |
| P5 | F | 11 to 20 | Malaysia | Pharmaceutical industry | Communicator |
| P6 | F | Above 20 | Malaysia | Pharmaceutical industry | Communicator |
| P7 | F | Above 20 | Malaysia | Pharmaceutical industry | Communicator |
| P8 | F | 11 to 20 | Malaysia | Pharmacy Services Programme | Pharmacist |
| P9 | F | 6 to 10 | Malaysia | Pharmacy Services Programme | Pharmacist |
| P10 | F | 11 to 20 | Malaysia | Pharmacy Services Programme | Pharmacist |
| P11 | F | 11 to 20 | Malaysia | Private hospital | Pharmacist |
| P12 | F | 11 to 20 | Malaysia | Public hospital | Pharmacist |
| P13 | F | Above 20 | Malaysia | MOH Administrator/ policy maker | Pharmacist |
| P14 | M | 6 to 10 | Malaysia | Public hospital | Pharmacist |
| P15 | F | Above 20 | Malaysia | MOH Administrator/ policy maker | Pharmacist |
| P16 | F | Above 20 | Malaysia | MOH Administrator/ policy maker | Pharmacist |
| P17 | F | 11 to 20 | Malaysia | Pharmaceutical industry | Pharmacist |
| P18 | F | Above 20 | Malaysia | Pharmaceutical industry | Pharmacist |
| P19 | M | 11 to 20 | Malaysia | University hospital | Doctor |
| P20 | M | 11 to 20 | Malaysia | Public health consultant | Doctor |
| P21 | M | Above 20 | Malaysia | Public hospital | Doctor |
| P22 | M | Above 20 | Malaysia | Public hospital | Doctor |
| P23 | M | 11 to 20 | Malaysia | Public hospital | Doctor |
| P24 | F | 11 to 20 | Sweden | Pharmacovigilance consultant | Doctor |
| P25 | F | Above 20 | Global (Netherlands) | Regulatory | Pharmacist |
| P26 | M | Above 20 | Italy | University hospital | Pharmacist |
| P27 | F | Above 20 | Sweden | Communication consultant | Communicator |
| P28 | F | 11 to 20 | Global (Malaysia) | WHO | Doctor |
| P29 | F | 11 to 20 | Global (Philippines) | WHO | Doctor |
| P30 | F | 11 to 20 | Global (Oman) | WHO | Communicator |
| P31 | M | 11 to 20 | Global (Switzerland) | WHO | Pharmacist |
| P32 | F | 11 to 20 | Global (Switzerland) | WHO | Communicator |
| P33 | M | Above 20 | Global (Switzerland) | WHO | Doctor |
| P34 | F | 11 to 20 | Australia | Regulatory | Doctor |
| P35 | F | 11 to 20 | Australia | Regulatory | Pharmacist |
| P36 | F | 11 to 20 | Australia | Regulatory | Communicator |
| P37 | F | 11 to 20 | Singapore | Regulatory | Pharmacist |
| P38 | F | 11 to 20 | Singapore | Regulatory | Pharmacist |
| P39 | F | 11 to 20 | Singapore | Regulatory | Pharmacist |
| P40* | M | 11 to 20 | Malaysia | Private hospital | Doctor |
| P41* | F | Above 20 | Malaysia | Public hospital | Doctor |
| P42* | F | 6 to 10 | Malaysia | Pharmaceutical industry | Communicator |

*Non-responder in Round 2

MOH: Ministry of Health; WHO: World Health Organisation

**Supplementary Table S4** List of strategies for Round 2 of the Delphi study

|  | **Strategy** |
| --- | --- |
| **Domain 1: Improve the format and content of medicines risk communication** | |
| 1.1 | Use a standardised format for medicines risk communication to make it easily recognisable. |
| 1.2 | More concise communication, e.g. increased use of infographics with links to full details. |
| 1.3 | Create multi-lingual communication for consumers (e.g. English and Malay language). |
| 1.4 | **New: Be transparent and communicate uncertainties (e.g. if information is still under assessment).* |
| **Domain 2: Implement educational programmes to increase awareness on medicines safety and regulatory risk communication** | |
| 2.1 | Conduct **regular** continuing medical education (CME) sessions for healthcare professionals. |
| 2.2 | Increase **outreach** of educational programmes or CME (e.g. to private sector healthcare professionals). |
| 2.3 | Develop online videos on medicines safety to increase awareness among **consumers**. |
| 2.4 | Incorporate medicines safety information in training programmes for **newly qualified** healthcare professionals (e.g. medical houseofficers, provisionally-registered pharmacists). |
| 2.5 | Develop module on medicines safety for **undergraduates** (health science courses) |
| 2.6 | **New: Conduct educational programmes using social media or existing popular platforms, websites and apps.* |
| 2.7 | **New: Initiate educational programmes on medicines safety as early or upstream as possible (e.g. among school children).* |
| 2.8 | **New: Organise medicines safety events at hospitals and clinics for healthcare professionals or consumers.* |
| **Domain 3: Translate and integrate risk communication information into practice** | |
| 3.1 | Release **official directives** from the Ministry of Health to enforce risk communication about important medicines safety changes. |
| 3.2 | Send out **letters from the NRA** directly to healthcare professionals to reinforce communication about high risk medicines safety issues. |
| 3.3 | Incorporate **pop-up safety alerts** into electronic prescribing systems (e.g. new interactions or contraindications). |
| 3.4 | Incorporate **medicines safety recommendations** into electronic prescribing systems (e.g. counselling points). |
| 3.5 | **New: Highlight important medicines safety updates at the beginning of the product prescribing information (PI).* |
| 3.6 | **New: Establish a process for accountability (e.g. a system to show that healthcare professionals have read and integrated risk communication information into practice).* |
| 3.7 | **New: Offer incentives (e.g. rating points or tax rebates) to pharmaceutical companies which maintain effective medicines risk communication systems.* |
| **Domain 4: Increase the use of technology in medicines risk communication** | |
| 4.1 | Improve effectiveness of NRA website (e.g. create user-friendly interface, feedback form for risk communication) |
| 4.2 | Establish and maintain **social media** tools for communicating general medicines safety information (especially with the public). |
| 4.3 | Maintain a data repository of medicines safety issues (e.g. make it searchable, allow public access, include practice recommendations) |
| 4.4 | **New: Leverage Artificial Intelligence tools (e.g., chatbot, videos) to make searching easier and generate more interactive communication.* |
| **Domain 5: Evaluate the effectiveness of medicines risk communication** | |
| 5.1 | Assess the effectiveness of social media tools in reaching target audiences. |
| 5.2 | Assess the reading level and language used in public communication to ensure it is appropriate for the target audience. |
| 5.3 | **New: Provide link to a feedback form for every communication sent out.* |
| **Domain 6: Increase collaboration to improve content and widen dissemination of medicines risk communication.**  Consider the collaboration of an NRA with the following stakeholders. | |
| 6.1 | Government organisations (e.g. Ministry of Health state directors, directors of hospitals and heads of departments assist in disseminating NRA communication to their staff) |
| 6.2 | Clinicians (e.g. practising doctors, pharmacists, dentists and nurses contribute articles on medicines safety or collaborate with NRA in research) |
| 6.3 | Pharmaceutical industry (e.g. collaborate in research, contribute articles and disseminate NRA communication) |
| 6.4 | Professional associations (e.g. Malaysian Medical Association, Malaysian Pharmacists Society, Malaysian Dental Association, Malaysian Nurses Association: contribute articles and disseminate NRA communication) |
| 6.5 | Consumer or patient organisations (e.g. contribute articles and disseminate NRA communication) |
| 6.6 | Owners of existing websites or apps regularly used by consumers or healthcare professionals to obtain medicines safety information (e.g. link important NPRA safety information directly through existing websites such as MiMS) |
| 6.7 | **New: International medicines safety bodies (e.g. collaborate with the World Health Organisation, vaccine safety communication network, or other NRAs).* |
| 6.8 | **New: Undergraduates or student healthcare professionals (e.g. collaborate in research).* |

*** new strategies suggested by panelists in Round 1

CME: continuing medical education; NPRA: National Pharmaceutical Regulatory Agency; NRA: national regulatory agency

**Supplementary Table S5** List of priority domains and strategies following Round 2 of the Delphi survey

|  | **Domains in order of priority** | **Mean priority score in Round 2** | |
| --- | --- | --- | --- |
| 1 | Improve the format and content of medicines risk communication | 4.21 |  |
| 2 | Increase the use of technology in medicines risk communication | 3.98 |  |
| 3 | Increase collaboration to improve content and widen dissemination of medicines risk communication. | 3.95 |  |
| 4 | Translate and integrate risk communication information into practice | 3.84 |  |
| 5 | Implement educational programmes to increase awareness on medicines safety and regulatory risk communication | 3.80 |  |
| 6 | Evaluate the effectiveness of medicines risk communication | 3.75 |  |
|  |  |  |  |

|  | **Strategies in order of priority** | **% rated 4 or 5**  **in Round 2** |
| --- | --- | --- |
| 1 | More concise communication, e.g. increased use of infographics with links to full details. | 94.9 |
| 2 | Collaboration with professional associations (e.g. Malaysian Medical Association, Malaysian Pharmacists Society, Malaysian Dental Association, Malaysian Nurses Association: contribute articles and disseminate NRA communication) | 94.9 |
| 3 | Incorporate medicines safety information in training programmes for newly qualified healthcare professionals (e.g. medical house officers, provisionally-registered pharmacists). | 94.9 |
| 4 | Collaboration with clinicians (e.g. practising doctors, pharmacists, dentists and nurses contribute articles on medicines safety or collaborate with NRA in research) | 92.3 |
| 5 | Create multi-lingual communication for consumers (e.g. English and Malay language). | 87.2 |
| 6 | Release official directives from the Ministry of Health to enforce risk communication about important medicines safety changes. | 87.2 |
| 7 | Maintain a data repository of medicines safety issues (e.g. make it searchable, allow public access, include practice recommendations) | 84.6 |
| 8 | Collaboration with government organisations (e.g. Ministry of Health state directors, directors of hospitals and heads of departments assist in disseminating NRA communication to their staff) | 84.6 |
| 9 | Collaboration with international medicines safety bodies (e.g. the World Health Organisation, vaccine safety communication network, or other NRAs). | 84.6 |
| 10 | Incorporate medicines safety recommendations into electronic prescribing systems (e.g. counselling points). | 84.6 |
| 11 | Assess the reading level and language used in public communication to ensure it is appropriate for the target audience. | 84.6 |
| 12 | Use a standardised format for medicines risk communication to make it easily recognisable. | 82.1 |
| 13 | Be transparent and communicate uncertainties (e.g. information is still under assessment). | 82.1 |
| 14 | Send out letters from the NRA directly to healthcare professionals to reinforce communication about high risk medicines safety issues. | 82.1 |
| 15 | Improve effectiveness of NRA website (e.g. create user-friendly interface, feedback form for risk communication) | 79.5 |
| 16 | Collaboration with pharmaceutical industry (e.g. collaborate in research, contribute articles and disseminate NRA communication) | 79.5 |
| 17 | Collaboration with owners of existing websites or apps regularly used by consumers or healthcare professionals to obtain medicines safety information (e.g. link important NRA safety information directly through existing websites such as MiMS) | 79.5 |
| 18 | Incorporate pop-up safety alerts into electronic prescribing systems (e.g. new interactions or contraindications). | 79.5 |
| 19 | Develop module on medicines safety for undergraduates (health science courses) | 79.5 |
| 20 | Establish and maintain social media tools for communicating general medicines safety information (especially with the public). | 76.5 |
| 21 | Collaboration with Consumer or patient organisations (e.g. contribute articles and disseminate NRA communication) | 76.5 |

NRA: national regulatory agency

**Supplementary Table S6** Data analysis (Round 1 and 2 of the Delphi study)

|  | **Strategies** | **Round 1** | | **Round 2** | | | | | | | |
| --- | --- | --- | --- | --- | --- | --- | --- | --- | --- | --- | --- |
|  |  | **Mean** | **SD** | **Mean** | **SD** | **% rated 4 or 5** | **Disagreement (% rated**  **1 or 2)** | **Communicators vs. recipients** | | **Local vs. international** | |
|  |  |  |  |  |  |  |  | **χ^2^** | **p-value** | **χ^2^** | **p-value** |
|  | **Domain 1: Improve the format and content of medicines risk communication** | 3.75 |  | 4.21 |  |  |  |  |  |  |  |
| 1.1 | Use a standardised format for medicines risk communication to make it easily recognisable. | 3.91 | 0.93 | 4.05 | 0.72 | 82.1 | 2.6 | 0.64 | 0.887 | 1.03 | 0.795 |
| 1.2 | More concise communication, e.g. increased use of infographics with links to full details. | 4.38 | 0.70 | 4.49 | 0.60 | 94.9 | 0.0 | 2.36 | 0.308 | 2.95 | 0.229 |
| 1.3 | Create multi-lingual communication for consumers (e.g. English and Malay language). | 4.33 | 0.85 | 4.21 | 0.66 | 87.2 | 0.0 | 4.49 | 0.106 | 0.95 | 0.624 |
| 1.4 | New: Be transparent and communicate uncertainties (e.g. information is still under assessment). |  |  | 4.10 | 0.75 | 82.1 | 2.6 | 2.85 | 0.415 | 2.84 | 0.418 |
|  | D**omain 2: Implement educational programmes to increase awareness on medicines safety and regulatory risk communication** | 3.93 |  | 3.80 |  |  |  |  |  |  |  |
| 2.1 | Conduct regular continuing medical education (CME) sessions for healthcare professionals | 3.91 | 0.93 | 3.85 | 0.59 | 74.4 | 0.0 | 0.57 | 0.752 | 0.73 | 0.695 |
| 2.2 | Increase outreach of educational programmes or CME (e.g. to private sector healthcare professionals). | 3.88 | 0.89 | 3.87 | 0.66 | 71.8 | 0.0 | 1.98 | 0.372 | **6.44** | **0.040*** |
| 2.3 | Develop online videos on medicines safety to increase awareness among consumers. | 3.88 | 0.83 | 3.69 | 0.66 | 59.0 | 0.0 | 4.54 | 0.103 | 3.15 | 0.207 |
| 2.4 | Incorporate medicines safety information in training programmes for newly qualified healthcare professionals (e.g. medical houseofficers, provisionally-registered pharmacists). | 4.26 | 0.80 | 4.36 | 0.58 | 94.9 | 0.0 | 0.59 | 0.745 | 0.18 | 0.915 |
| 2.5 | Develop module on medicines safety for undergraduates (health science courses) | 4.07 | 0.87 | 4.18 | 0.82 | 79.5 | 2.6 | 1.65 | 0.648 | 1.54 | 0.674 |
| 2.6 | New: Conduct educational programmes using social media or existing popular platforms, websites and apps. |  |  | 3.77 | 0.78 | 66.7 | 5.1 | 4.38 | 0.223 | 7.11 | 0.068 |
| 2.7 | New: Initiate educational programmes on medicines safety as early or upstream as possible (e.g. among school children). |  |  | 3.18 | 1.05 | 35.9 | 28.2 | 0.69 | 0.953 | 4.22 | 0.377 |
| 2.8 | New: Organise medicines safety events at hospitals and clinics for healthcare professionals or consumers. |  |  | 3.46 | 0.76 | 46.2 | 7.7 | 2.80 | 0.423 | 5.75 | 0.124 |
|  | **Domain 3: Translate and integrate risk communication information into practice** | 4.01 |  | 3.84 |  |  |  |  |  |  |  |
| 3.1 | Release official directives from the Ministry of Health to enforce risk communication about important medicines safety changes. | 4.12 | 0.74 | 4.13 | 0.62 | 87.2 | 0.0 | 0.60 | 0.741 | 1.25 | 0.535 |
| 3.2 | Send out letters from the NRA directly to healthcare professionals to reinforce communication about high risk medicines safety issues. | 3.79 | 0.87 | 3.92 | 0.70 | 82.1 | 5.1 | 1.12 | 0.772 | 6.27 | 0.099 |
| 3.3 | Incorporate pop-up safety alerts into electronic prescribing systems (e.g. new interactions or contraindications). | 4.02 | 1.00 | 4.18 | 0.76 | 79.5 | 0.0 | 2.79 | 0.247 | 0.36 | 0.837 |
| 3.4 | Incorporate medicines safety recommendations into electronic prescribing systems (e.g. counselling points). | 4.12 | 0.92 | 4.08 | 0.62 | 84.6 | 0.0 | 1.25 | 0.535 | 2.26 | 0.323 |
| 3.5 | New: Highlight important medicines safety updates at the beginning of the product prescribing information (PI). |  |  | 3.92 | 0.87 | 74.4 | 7.7 | 2.19 | 0.533 | 2.08 | 0.555 |
| 3.6 | New: Establish a process for accountability (e.g. a system to show that healthcare professionals have read and integrated risk communication information into practice). |  |  | 3.69 | 0.83 | 61.5 | 7.7 | 0.63 | 0.891 | 1.46 | 0.692 |
| 3.7 | New: Offer incentives (e.g. rating points or tax rebates) to pharmaceutical companies which maintain effective medicines risk communication systems. |  |  | 2.95 | 1.08 | 33.3 | 33.3 | **10.11** | **0.039** | **14.26** | **0.007** |
|  | **Domain 4: Increase the use of technology in medicines risk communication** | 3.97 |  | 3.98 |  |  |  |  |  |  |  |
| 4.1 | Improve effectiveness of NRA website (e.g. create user-friendly interface, feedback form for risk communication) | 4.41 | 0.83 | 4.26 | 0.91 | 79.5 | 5.1 | 2.16 | 0.540 | 1.34 | 0.719 |
| 4.2 | Establish and maintain social media tools for communicating general medicines safety information (especially with the public). | 4.07 | 0.71 | 3.97 | 0.74 | 76.9 | 2.6 | 1.25 | 0.741 | 4.35 | 0.226 |
| 4.3 | Maintain a data repository of medicines safety issues (e.g. make it searchable, allow public access, include practice recommendations) | 4.19 | 0.86 | 4.18 | 0.76 | 84.6 | 2.6 | 3.07 | 0.381 | 5.44 | 0.142 |
| 4.4 | New: Leverage Artificial Intelligence tools (e.g., chatbot, videos) to make searching easier and generate more interactive communication. |  |  | 3.51 | 1.00 | 48.7 | 12.8 | 4.59 | 0.333 | 1.00 | 0.040 |
|  | **Domain 5: Evaluate the effectiveness of medicines risk communication** | 3.77 |  | 3.75 |  |  |  |  |  |  |  |
| 5.1 | Assess the effectiveness of social media tools in reaching target audiences. | 3.83 | 0.82 | 3.80 | 0.70 | 69.2 | 2.6 | 2.31 | 0.512 | 1.73 | 0.631 |
| 5.2 | Assess the reading level and language used in public communication to ensure it is appropriate for the target audience. | 4.07 | 0.97 | 4.00 | 0.80 | 84.6 | 7.7 | 3.69 | 0.297 | 1.06 | 0.788 |
| 5.3 | New: Provide link to a feedback form for every communication sent out. |  |  | 3.462 | 0.884 | 46.15 | 12.82 | 1.897 | 0.594 | 0.089 | 0.993 |
|  | **Domain 6: Increase collaboration to improve content and widen dissemination of medicines risk communication.** | 3.925 |  | 3.946 |  |  |  |  |  |  |  |
| 6.1 | Collaboration with government organisations (e.g. Ministry of Health state directors, directors of hospitals and heads of departments assist in disseminating NRA communication to their staff) | 3.952 | 0.764 | 4.077 | 0.623 | 84.62 | 0.00 | 0.938 | 0.626 | 0.596 | 0.742 |
| 6.2 | Collaboration with clinicians (e.g. practising doctors, pharmacists, dentists and nurses contribute articles on medicines safety or collaborate with NRA in research) | 4.286 | 0.673 | 4.333 | 0.621 | 92.31 | 0.00 | 3.225 | 0.199 | 1.164 | 0.559 |
| 6.3 | Collaboration with pharmaceutical industry (e.g. collaborate in research, contribute articles and disseminate NRA communication) | 3.810 | 0.862 | 3.795 | 0.732 | 79.49 | 5.13 | 4.286 | 0.369 | 2.441 | 0.655 |
| 6.4 | Collaboration with professional associations (e.g. Malaysian Medical Association, Malaysian Pharmacists Society, Malaysian Dental Association, Malaysian Nurses Association: contribute articles and disseminate NRA communication) | 4.095 | 0.759 | 4.231 | 0.536 | 94.87 | 0.00 | 1.490 | 0.475 | 0.226 | 0.893 |
| 6.5 | Collaboration with consumer or patient organisations (e.g. contribute articles and disseminate NRA communication) | 3.881 | 0.942 | 3.923 | 0.703 | 76.92 | 2.56 | 1.057 | 0.787 | 3.072 | 0.381 |
| 6.6 | Collaboration with owners of existing websites or apps regularly used by consumers or healthcare professionals to obtain medicines safety information (e.g. link important NRA safety information directly through existing websites such as MiMS) | 3.976 | 0.811 | 3.974 | 0.707 | 79.49 | 2.56 | 2.892 | 0.409 | 2.084 | 0.555 |
| 6.7 | New: International medicines safety bodies (e.g. collaborate with the World Health Organisation, vaccine safety communication network, or other NRAs). |  |  | 3.949 | 0.510 | 84.62 | 0.00 | 0.142 | 0.931 | 0.448 | 0.799 |
| 6.8 | New: Undergraduates or student healthcare professionals (e.g. collaborate in research). |  |  | 3.282 | 0.724 | 28.21 | 7.69 | 3.083 | 0.379 | 6.451 | 0.092 |

Statistically significant p-values are shown in bold. CME: continuing medical education; NRA: national regulatory agency; SD: standard deviation.

**Supplementary Table S7** Subgroup analysis comparing the mean priority scores for each strategy in Round 2 between (i) communicators versus recipients and (ii) local versus international panellists

|  | **Strategies** | **Mean priority scores** | | | |
| --- | --- | --- | --- | --- | --- |
|  |  | **Communicators** | **Recipients** | **Local panellists** | **International panellists** |
| **Domain 1: Improve the format and content of medicines risk communication** | |  |  |  |  |
| 1.1 | Use a standardised format for medicines risk communication to make it easily recognisable. | 4.04 | 4.08 | 4.00 | 4.13 |
| 1.2 | More concise communication, e.g. increased use of infographics with links to full details. | 4.39 | 4.69 | 4.61 | 4.31 |
| 1.3 | Create multi-lingual communication for consumers (e.g. English and Malay language). | 4.19 | 4.23 | 4.26 | 4.13 |
| 1.4 | **New: Be transparent and communicate uncertainties (e.g. if information is still under assessment).* | 3.96 | 4.39 | 4.17 | 4.00 |
| **Domain 2: Implement educational programmes to increase awareness on medicine safety and regulatory risk communication** | |  |  |  |  |
| 2.1 | Conduct **regular** continuing medical education (CME) sessions for healthcare professionals. | 3.81 | 3.92 | 3.87 | 3.81 |
| 2.2 | Increase **outreach** of educational programmes or CME (e.g. to private sector healthcare professionals). | 3.77 | 4.08 | 4.04 | 3.63 |
| 2.3 | Develop online videos on medicines safety to increase awareness among **consumers**. | 3.65 | 3.77 | 3.78 | 3.56 |
| 2.4 | Incorporate medicines safety information in training programmes for **newly qualified** healthcare professionals (e.g. medical houseofficers, provisionally-registered pharmacists). | 4.35 | 4.39 | 4.39 | 4.31 |
| 2.5 | Develop module on medicines safety for **undergraduates** (health science courses) | 4.08 | 4.39 | 4.26 | 4.06 |
| 2.6 | **New: Conduct educational programmes using social media or existing popular platforms, websites and apps.* | 3.69 | 3.92 | 4.00 | 3.44 |
| 2.7 | **New: Initiate educational programmes on medicines safety as early or upstream as possible (e.g. among school children).* | 3.12 | 3.31 | 3.44 | 2.81 |
| 2.8 | **New: Organise medicines safety events at hospitals and clinics for healthcare professionals or consumers.* | 3.35 | 3.69 | 3.70 | 3.13 |
| **Domain 3: Translate and integrate risk communication information into practice** | |  |  |  |  |
| 3.1 | Release **official directives** from the Ministry of Health to enforce risk communication about important medicines safety changes. | 4.08 | 4.23 | 4.22 | 4.00 |
| 3.2 | Send out **letters from the NRA** directly to healthcare professionals to reinforce communication about high risk medicines safety issues. | 4.00 | 3.77 | 3.74 | 4.19 |
| 3.3 | Incorporate **pop-up safety alerts** into electronic prescribing systems (e.g. new interactions or contraindications). | 4.12 | 4.31 | 4.22 | 4.13 |
| 3.4 | Incorporate **medicines safety recommendations** into electronic prescribing systems (e.g. counselling points). | 4.00 | 4.23 | 4.13 | 4.00 |
| 3.5 | **New: Highlight important medicines safety updates at the beginning of the product prescribing information (PI).* | 3.96 | 3.85 | 3.91 | 3.94 |
| 3.6 | **New: Establish a process for accountability (e.g. a system to show that healthcare professionals have read and integrated risk communication information into practice).* | 3.65 | 3.77 | 3.74 | 3.63 |
| 3.7 | **New: Offer incentives (e.g. rating points or tax rebates) to pharmaceutical companies which maintain effective medicines risk communication systems.* | 2.65 | 3.54 | 3.48 | 2.19 |
| **Domain 4: Increase the use of technology in medicines risk communication** | |  |  |  |  |
| 4.1 | Improve effectiveness of NRA website (e.g. create user-friendly interface, feedback form for risk communication) | 4.31 | 4.15 | 4.30 | 4.19 |
| 4.2 | Establish and maintain **social media** tools for communicating general medicines safety information (especially with the public). | 3.89 | 4.15 | 4.17 | 3.69 |
| 4.3 | Maintain a data repository of medicines safety issues (e.g. make it searchable, allow public access, include practice recommendations) | 4.04 | 4.46 | 4.35 | 3.94 |
| 4.4 | **New: Leverage Artificial Intelligence tools (e.g., chatbot, videos) to make searching easier and generate more interactive communication.* | 3.46 | 3.62 | 3.83 | 3.06 |
| **Domain 5: Evaluate the effectiveness of medicines risk communication** | |  |  |  |  |
| 5.1 | Assess the effectiveness of social media tools in reaching target audiences. | 3.81 | 3.77 | 3.83 | 3.75 |
| 5.2 | Assess the reading level and language used in public communication to ensure it is appropriate for the target audience. | 4.00 | 4.00 | 4.00 | 4.00 |
| 5.3 | **New: Provide link to a feedback form for every communication sent out.* | 3.46 | 3.46 | 3.48 | 3.44 |
| **Domain 6: Increase collaboration to improve content and widen dissemination of medicines risk communication.**  Consider the collaboration of an NRA with the following stakeholders. | |  |  |  |  |
| 6.1 | Government organisations (e.g. Ministry of Health state directors, directors of hospitals and heads of departments assist in disseminating NRA communication to their staff) | 4.12 | 4.00 | 4.09 | 4.06 |
| 6.2 | Clinicians (e.g. practising doctors, pharmacists, dentists and nurses contribute articles on medicines safety or collaborate with NRA in research) | 4.35 | 4.31 | 4.35 | 4.31 |
| 6.3 | Pharmaceutical industry (e.g. collaborate in research, contribute articles and disseminate NRA communication) | 3.77 | 3.85 | 3.87 | 3.69 |
| 6.4 | Professional associations (e.g. Malaysian Medical Association, Malaysian Pharmacists Society, Malaysian Dental Association, Malaysian Nurses Association: contribute articles and disseminate NRA communication) | 4.23 | 4.23 | 4.22 | 4.25 |
| 6.5 | Consumer or patient organisations (e.g. contribute articles and disseminate NRA communication) | 3.85 | 4.08 | 3.87 | 4.00 |
| 6.6 | Owners of existing websites or apps regularly used by consumers or healthcare professionals to obtain medicines safety information (e.g. link important NPRA safety information directly through existing websites such as MiMS) | 4.00 | 3.92 | 4.00 | 3.94 |
| 6.7 | **New: International medicines safety bodies (e.g. collaborate with the World Health Organisation, vaccine safety communication network, or other NRAs).* | 3.96 | 3.92 | 3.96 | 3.94 |
| 6.8 | **New: Undergraduates or student healthcare professionals (e.g. collaborate in research).* | 3.15 | 3.54 | 3.48 | 3.00 |

*** new strategies suggested by panelists in Round 1

CME: continuing medical education; NPRA: National Pharmaceutical Regulatory Agency; NRA: national regulatory agency

Key:

strategies evaluated as high priority for each comparison group
