# Supplementary material for: Strategies to enhance risk communication about medicines in Malaysia: a Delphi study among multinational experts
Source: BMC Health Serv Res. 2024 Sep 3;24:1019. doi: 10.1186/s12913-024-11476-0 (PMC11373486; doi:10.1186/s12913-024-11476-0)
Supplement: Supplementary file 2 — Supplementary Material 2. Additional file 2: Round 1 Delphi questionnaire [file 12913_2024_11476_MOESM2_ESM.pdf]

Additional file 2: Round 1 Delphi Questionnaire

Medication Risk Communication Delphi Study (Round 1)

Thank you for considering to participate in this study.

This Delphi study aims to establish consensus on the list of strategies to enhance medication risk communication by regulatory agencies. The outcome of this study will be used to develop a 5-year strategic plan for medication risk communication in Malaysia.

You are invited to participate in this study because you are a key stakeholder in medication risk communication, either as a communication expert, clinician, pharmacist, researcher or policy maker.

We compiled a list of strategies to enhance risk communication, which have been classified into six domains as follow:
Communication essentials Educational programs Integration into practice Using technology Evaluation of effectiveness Increasing collaboration In Round 1 of this study, we ask you to rate each strategy in terms of priority for implementation by national regulatory agencies (NRAs) such as the National Pharmaceutical Regulatory Agency (NPRA), Malaysia. You may also suggest additional strategies and leave comments.
We greatly appreciate your time and opinion.

Thank you and with best regards,

[Investigator name and designation]

[Supervisors names and designations]

General Instructions: This survey will take approximately 10-15 minutes to complete. Your participation is voluntary. By clicking "Yes, I consent" below, you indicate that you have fully read and understood the Participant Information Sheet provided.
For further details on the study, please refer to the Participant Information Sheet. To view samples of NPRA risk communication, please click here. If you have any questions please contact: *[Investigator name]* at *[email address]*.
We sincerely thank you for your participation.

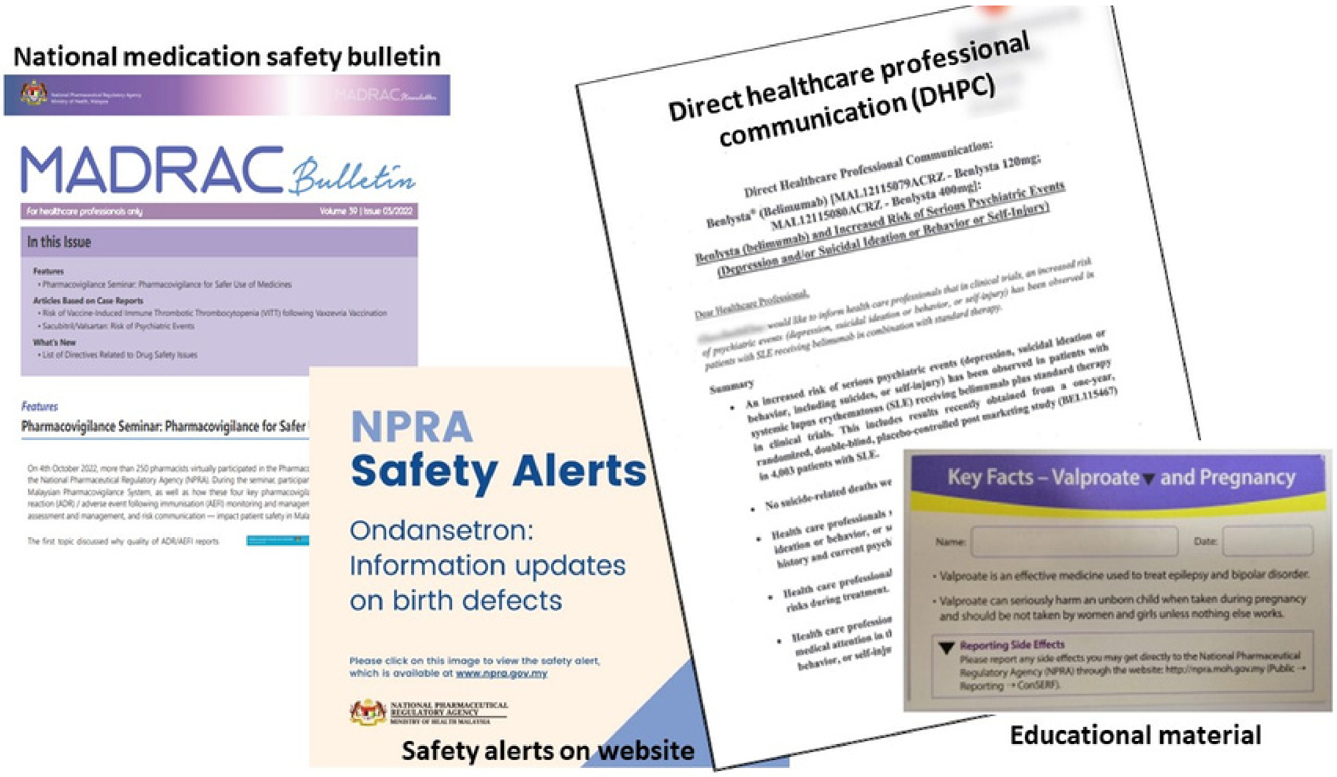

Participant consent

By checking this box, I hereby agree to participate in this study.

☐ Yes, I consent

SECTION 1: Demographic information

Name

____

Gender

◯ Female
◯ Male

Age (years)

____

Please indicate your designation:

- ◯ Communicator
- ◯ Dentist
- ◯ Doctor
- ◯ Nurse
- ◯ Pharmacist
- ◯ Other (please specify)

Please specify Other designation: __________________________________

Please indicate your field of expertise (select all that apply)

- ☐ Academia
- ☐ Administrative
- ☐ Communication
- ☐ Dental
- ☐ Healthcare innovations
- ☐ Medicine
- ☐ Nursing
- ☐ Pharmacy
- ☐ Public health
- ☐ Regulatory affairs
- ☐ Research
- ☐ Others (please specify)

Please enter Other fields of expertise (if relevant): __________________________________

Please indicate the total years of experience in your field of expertise:

- ◯ Below 3 years
- ◯ 3 to 5 years
- ◯ 6 to 10 years
- ◯ 11 to 20 years
- ◯ Above 20 years

Please indicate your current main place of work:

- ◯ Malaysia
- ◯ Australia
- ◯ Italy
- ◯ Netherlands
- ◯ Oman
- ◯ Singapore
- ◯ Sweden
- ◯ United Kingdom
- ◯ Other (please specify)

Please specify Other main place of work (country): __________________________________

SECTION 2: Prioritising strategies

Domain 1: Communication essentials- improve the format and content of medication risk communication

Please rate each strategy in terms of priority for implementation by national regulatory agencies (NRAs).

	Not a priority	Low priority	Medium priority	High priority	Highest priority
Use a standardised format for medication risk communication to make it easily recognisable.	○	○	○	○	○
More concise communication, e.g. increased use of infographics with links to full details.	○	○	○	○	○
Increase use of narrative-style messages e.g. storytelling to explain a case study	○	○	○	○	○
Increase original content e.g. local data or case studies	○	○	○	○	○
Make the communication more engaging, e.g. 1 minute videos with links to full details.	○	○	○	○	○
Create templates of risk communication messages for different situations (e.g. traffic light colour coding based on level of risk)	○	○	○	○	○
Hire staff or external experts with required expertise (e.g. communication experts, graphic designers, social media managers)	○	○	○	○	○
Create multi-lingual communication for consumers (e.g. English and Malay language).	○	○	○	○	○
Establish an external medication risk communication advisory board to review regulatory risk communication.	○	○	○	○	○

If you can think of additional strategies to improve the format and content of medication risk communication other than those listed in this questionnaire, please list them here: ____________________

Domain 2: Implement educational programmes to increase awareness on medication safety and regulatory risk communication

Please rate each strategy in terms of priority for implemention by NRAs or other appropriate bodies.

	Not a priority	Low priority	Medium priority	High priority	Highest priority
Conduct regular continuing medical education (CME) sessions for healthcare professionals	○	○	○	○	○
Conduct online CME sessions for healthcare professionals.	○	○	○	○	○
Increase outreach of educational programmes or CME (e.g. to private sector healthcare professionals).	○	○	○	○	○
Develop online videos on medication safety for self-guided learning by healthcare professionals.	○	○	○	○	○
Develop online videos on medication safety to increase awareness among consumers.	○	○	○	○	○
Incorporate medication safety information in training programmes for newly qualified healthcare professionals (e.g. medical houseofficers, provisionally-registered pharmacists).	○	○	○	○	○
Develop module on medication safety for undergraduates (health science courses)	○	○	○	○	○

If you can think of additional strategies to improve educational programmes related to medication risk communication, please list them here: ________________________________

Domain 3: Translate and integrate risk communication information into practice

Please rate each strategy in terms of priority for implemention by NRAs or other appropriate bodies.

	Not a priority	Low priority	Medium priority	High priority	Highest priority

Release official directives from the Ministry of Health to enforce risk communication on important medication safety changes.	○	○	○	○	○
Send out letters from the NRA directly to healthcare professionals to reinforce communication on high risk medication safety issues.	○	○	○	○	○
Incorporate pop-up safety alerts into electronic prescribing systems (e.g. new interactions or contraindications).	○	○	○	○	○
Incorporate medication safety recommendations into electronic prescribing systems (e.g. counselling points).	○	○	○	○	○

If you can think of additional strategies to integrate medication risk communication information into practice, please list them here: ____

Domain 4: Increase the use of technology in medication risk communication

Please rate each strategy in terms of priority for implemention by NRAs.

	Not a priority	Low priority	Medium priority	High priority	Highest priority
Improve effectiveness of NRA website (e.g. create user-friendly interface, feedback form for risk communication)	○	○	○	○	○
Allow users to specify which area of interest they would like to receive medication risk communication emails.	○	○	○	○	○
Establish and maintain social media tools for communicating general medication safety information (especially with the public).	○	○	○	○	○

Use a mobile phone application for medication risk communication (e.g. allow users to receive targeted or personalised updates)	◯	◯	◯	◯	◯
Maintain a data repository of medication safety issues (e.g. make it searchable, allow public access, include practice recommendations)	◯	◯	◯	◯	◯

If you can think of additional strategies to increase the use of technology in medication risk communication, please list them here: ____

Domain 5: Evaluate the effectiveness of medication risk communication

Please rate each strategy in terms of priority for implementation by NRAs.

	Not a priority	Low priority	Medium priority	High priority	Highest priority
Review detailed breakdown of NRA website usage statistics.	◯	◯	◯	◯	◯
Assess the effectiveness of social media tools in reaching target audiences.	◯	◯	◯	◯	◯
Assess the reading level and language used in public communication to ensure it is appropriate for the target audience.	◯	◯	◯	◯	◯
Develop and conduct collaborative research on the impact of risk communication on healthcare professionals (e.g. changes in prescribing practice, ADR reporting rates, knowledge and awareness).	◯	◯	◯	◯	◯
Develop and conduct collaborative research on the impact of risk communication on consumers (e.g. awareness, knowledge and reported behaviours).	◯	◯	◯	◯	◯

If you can think of additional strategies to evaluate the effectiveness of medication risk communication, please list them here: ____

Domain 6: Increase collaboration to improve content and widen dissemination of medication risk communication.

Consider the collaboration of an NRA with the following stakeholders.
Please rate each strategy in terms of priority for implemention by NRAs.

	Not a priority	Low priority	Medium priority	High priority	Highest priority
Government organisations (e.g. Ministry of Health state directors, directors of hospitals and heads of departments assist in disseminating NRA communication to their staff)	○	○	○	○	○
Clinicians (e.g. practising doctors, pharmacists, dentists and nurses contribute articles on medication safety or collaborate with NRA in research)	○	○	○	○	○
Academicians (e.g. collaborate in research, contribute articles and disseminate NRA communication)	○	○	○	○	○
Pharmaceutical industry (e.g. collaborate in research, contribute articles and disseminate NRA communication)	○	○	○	○	○
Professional associations (e.g. Malaysian Medical Association, Malaysian Pharmacists Society, Malaysian Dental Association, Malaysian Nurses Association: contribute articles and disseminate NRA communication)	○	○	○	○	○
Consumer or patient organisations (e.g. contribute articles and disseminate NRA communication)	○	○	○	○	○
Owners of existing websites or apps regularly used by consumers or healthcare professionals to obtain medication safety information (e.g. link important NRA safety information directly through existing websites such as MiMS)	○	○	○	○	○

If you can think of additional strategies to increase collaboration for improving medication risk communication, please list them here: ____

SECTION 3: General comments

Please let us know any further comments you may have on the strategies to enhance regulatory agency medication risk communication: ____

End of questionnaire.

Thank you very much.
