# Supplementary material for: Strategies to enhance risk communication about medicines in Malaysia: a Delphi study among multinational experts
Source: BMC Health Serv Res. 2024 Sep 3;24:1019. doi: 10.1186/s12913-024-11476-0 (PMC11373486; doi:10.1186/s12913-024-11476-0)
Supplement: Supplementary file 3 — Supplementary Material 3. Additional file 3: Round 2 Delphi questionnaire [file 12913_2024_11476_MOESM3_ESM.pdf]

Medication Risk Communication Delphi Study (Round 2)

Dear Panelist,

Welcome to Round 2 of this Delphi study. Thank you for your continued participation and valuable input.
This modified Delphi study aims to establish consensus on the list of strategies to enhance medication risk communication in Malaysia.
Our Delphi panel comprises:
Communicators from Malaysian and international regulatory agencies, pharmaceutical industry, and public health bodies; Recipients of regulatory communication Malaysian doctors and pharmacists Based on the panel responses in Round 1, we have condensed the list of strategies and added some new strategies proposed by the panel. You will be informed of the average group rating for each strategy and asked to rate the priority of implementation once again.
We greatly appreciate your time and opinions.

Thank you and with best regards,

[Investigator name and designation]

[Supervisors names and designations]

General Instructions:
This survey will take approximately 10-15 minutes to complete.
In this round, you will be provided with the average group rating for each strategy from Round 1. Considering this, please rate the priority of implementation once again.
*New strategies are listed in italics.
For further details on the study, please refer to the Participant Information Sheet or video. To view samples of NPRA risk communication, please click here. If you have any questions please contact: *[Name]* (main researcher) by sending an email to *[email address]*.

We sincerely thank you for your participation.

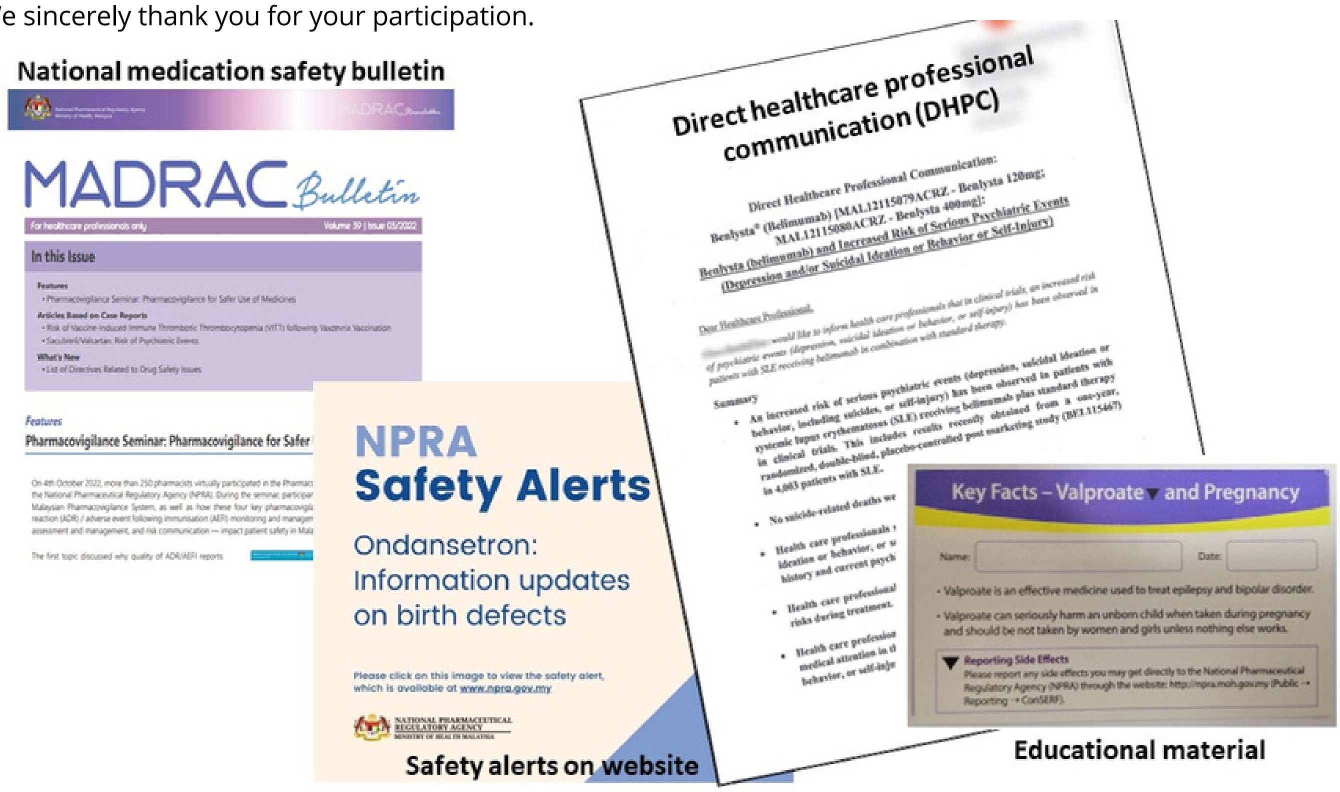

Participant consent

By checking this box, I hereby agree to participate in this study.

☐ Yes, I consent

SECTION 1: Demographic information

Name

____

SECTION 2: Prioritising strategies

You are provided with the average group rating for each strategy from Round 1, and asked to rate the priority of implementation once again.
*New strategies are listed in italic font.

Domain 1: Improve the format and content of medication risk communication

Please rate each strategy in terms of priority for implementation by national regulatory agencies (NRAs).

1.1 Use a standardised format for NPRA medication risk communication to make it easily recognisable.

(Round 1: Your rating= 5, Average group rating= 3.90)

- ◯ 1- not a priority
- ◯ 2- low priority
- ◯ 3- medium priority
- ◯ 4- high priority
- ◯ 5- highest priority

1.2 More concise communication, e.g. increased use of infographics with links to full details.

(Round 1: Your rating= 4, Average group rating: 4.38)

- ◯ 1- not a priority
- ◯ 2- low priority
- ◯ 3- medium priority
- ◯ 4- high priority
- ◯ 5- highest priority

1.3 Create multi-lingual communication for consumers (e.g. English and Malay language).

(Round 1: Your rating= 3, Average group rating= 4.33)

- ◯ 1- not a priority
- ◯ 2- low priority
- ◯ 3- medium priority
- ◯ 4- high priority
- ◯ 5- highest priority

1.4 Be transparent and communicate uncertainties (e.g. information is still under assessment).
(Newly added strategy)

- ◯ 1- not a priority
- ◯ 2- low priority
- ◯ 3- medium priority
- ◯ 4- high priority
- ◯ 5- highest priority

Domain 2: Implement educational programmes to increase awareness on medication safety and regulatory risk communication

Please rate each strategy in terms of priority for implemention by NRAs or other appropriate bodies.

2.1 Conduct regular continuing medical education (CME) sessions for healthcare professionals.

(Round 1: Your rating= 5, Average group rating= 3.90)

- ◯ 1- not a priority
- ◯ 2- low priority
- ◯ 3- medium priority
- ◯ 4- high priority
- ◯ 5- highest priority

2.2 Increase outreach of educational programmes or CME (e.g. to private sector healthcare professionals).

(Round 1: Your rating= 4, Average group rating= 3.88)

- ◯ 1- not a priority
- ◯ 2- low priority
- ◯ 3- medium priority
- ◯ 4- high priority
- ◯ 5- highest priority

2.3 Develop online videos on medication safety to increase awareness among consumers.

(Round 1: Your rating= 3, Average group rating= 3.88)

- ◯ 1- not a priority
- ◯ 2- low priority
- ◯ 3- medium priority
- ◯ 4- high priority
- ◯ 5- highest priority

2.4 Incorporate medication safety information in training programmes for newly qualified healthcare professionals (e.g. medical house officers, provisionally-registered pharmacists).
(Round 1: Your rating= 4, Average group rating= 4.26)

- ◯ 1- not a priority
- ◯ 2- low priority
- ◯ 3- medium priority
- ◯ 4- high priority
- ◯ 5- highest priority

2.5 Develop module on medication safety for undergraduates (health science courses).

(Round 1: Your rating= 4, Average group rating= 4.07)

- ◯ 1- not a priority
- ◯ 2- low priority
- ◯ 3- medium priority
- ◯ 4- high priority
- ◯ 5- highest priority

2.6 Conduct educational programmes using social media or existing popular platforms, websites and apps.
(New)

- ◯ 1- not a priority
- ◯ 2- low priority
- ◯ 3- medium priority
- ◯ 4- high priority
- ◯ 5- highest priority

2.7 Initiate educational programmes on medication safety as early or upstream as possible (e.g. among school children). (New)

- ◯ 1- not a priority
- ◯ 2- low priority
- ◯ 3- medium priority
- ◯ 4- high priority
- ◯ 5- highest priority

2.8 Organise medication safety events at hospitals and clinics for healthcare professionals or consumers.
(New)

- ◯ 1- not a priority
- ◯ 2- low priority
- ◯ 3- medium priority
- ◯ 4- high priority
- ◯ 5- highest priority

Domain 3: Translate and integrate risk communication information into practice

Please rate each strategy in terms of priority for implemention by NRAs or other appropriate bodies.

3.1 Release official directives from the Ministry of Health to enforce risk communication on important medication safety
changes.

(Round 1: Your rating= 5, Average group rating= 4.12)

- ◯ 1- not a priority
- ◯ 2- low priority
- ◯ 3- medium priority
- ◯ 4- high priority
- ◯ 5- highest priority

3.2 Send out letters from the NRA directly to healthcare professionals to reinforce communication on high-risk medication safety issues.
(Round 1: Your rating= 4, Average group rating= 3.79)

- ◯ 1- not a priority
- ◯ 2- low priority
- ◯ 3- medium priority
- ◯ 4- high priority
- ◯ 5- highest priority

3.3 Incorporate pop-up safety alerts into electronic prescribing systems (e.g. new interactions or contraindications).

(Round 1: Your rating= 4, Average group rating= 4.02)

- ◯ 1- not a priority
- ◯ 2- low priority
- ◯ 3- medium priority
- ◯ 4- high priority
- ◯ 5- highest priority

3.4 Incorporate medication safety recommendations into electronic prescribing systems (e.g. counselling points).

(Round 1: Your rating= 4, Average group rating= 4.12)

◯ 1- not a priority
◯ 2- low priority
◯ 3- medium priority
◯ 4- high priority
◯ 5- highest priority

3.5 Highlight important medication safety updates at the beginning of the product prescribing information (PI).
(New)

◯ 1- not a priority
◯ 2- low priority
◯ 3- medium priority
◯ 4- high priority
◯ 5- highest priority

3.6 Establish a process for accountability (e.g. a system to show that healthcare professionals have read and integrated risk communication information into practice).
(New)

◯ 1- not a priority
◯ 2- low priority
◯ 3- medium priority
◯ 4- high priority
◯ 5- highest priority

3.7 Offer incentives (e.g. rating points or tax rebates) to pharmaceutical companies which maintain effective medication risk communication systems.
(New)

◯ 1- not a priority
◯ 2- low priority
◯ 3- medium priority
◯ 4- high priority
◯ 5- highest priority

Domain 4: Increase the use of technology in medication risk communication

Please rate each strategy in terms of priority for implemention by NRAs.

4.1 Improve effectiveness of NRA website (e.g. create user-friendly interface, feedback form for risk communication).

(Round 1: Your rating= 5, Average group rating= 4.40)

◯ 1- not a priority
◯ 2- low priority
◯ 3- medium priority
◯ 4- high priority
◯ 5- highest priority

4.2 Establish and maintain social media tools for communicating general medication safety information (especially with the public).

(Round 1: Your rating= 3, Average group rating= 4.07)

- 1- not a priority
- 2- low priority
- 3- medium priority
- 4- high priority
- 5- highest priority

4.3 Maintain a data repository of medication safety issues (e.g. make it searchable, allow public access, include practice recommendations).

(Round 1: Your rating= 4, Average group rating= 4.19)

- 1- not a priority
- 2- low priority
- 3- medium priority
- 4- high priority
- 5- highest priority

4.4 Leverage Artificial Intelligence tools (e.g., chatbot, videos) to make searching easier and generate more interactive communication.
(New)

- 1- not a priority
- 2- low priority
- 3- medium priority
- 4- high priority
- 5- highest priority

Domain 5: Evaluate the effectiveness of medication risk communication

Please rate each strategy in terms of priority for implemention by NRAs.

5.1 Assess the effectiveness of social media tools in reaching target audiences.

(Round 1: Your rating= 5, Average group rating= 3.83)

- 1- not a priority
- 2- low priority
- 3- medium priority
- 4- high priority
- 5- highest priority

5.2 Assess the reading level and language used in public communication to ensure it is appropriate for the target audience.

(Round 1: Your rating= 4, Average group rating= 4.07)

- 1- not a priority
- 2- low priority
- 3- medium priority
- 4- high priority
- 5- highest priority

5.3 Provide link to a feedback form for every communication sent out.
(New)

- 1- not a priority
- 2- low priority
- 3- medium priority
- 4- high priority
- 5- highest priority

Domain 6: Increase collaboration to improve content and widen dissemination of medication risk communication.

Consider the collaboration of an NRA with the following stakeholders.
Please rate each strategy in terms of priority for implemention by NRAs.

6.1 Government organisations (e.g. Ministry of Health state directors, directors of hospitals and heads of departments assist in disseminating NRA communication to their staff).

(Round 1: Your rating= 3, Average group rating= 3.95)

- 1- not a priority
- 2- low priority
- 3- medium priority
- 4- high priority
- 5- highest priority

6.2 Clinicians (e.g. practising doctors, pharmacists, dentists and nurses contribute articles on medication safety or collaborate with NRA in research).
(Round 1: Your rating= 5, Average group rating= 4.29)

- 1- not a priority
- 2- low priority
- 3- medium priority
- 4- high priority
- 5- highest priority

6.3 Pharmaceutical industry (e.g. collaborate in research, contribute articles and disseminate NRA communication).

(Round 1: Your rating= 5, Average group rating= 3.81)

- 1- not a priority
- 2- low priority
- 3- medium priority
- 4- high priority
- 5- highest priority

6.4 Professional associations (e.g. Malaysian Medical Association, Malaysian Pharmacists Society: contribute articles and disseminate NRA communication).

(Round 1: Your rating= 4, Average group rating= 4.10)

- ◯ 1- not a priority
- ◯ 2- low priority
- ◯ 3- medium priority
- ◯ 4- high priority
- ◯ 5- highest priority

6.5 Consumer or patient organisations (e.g. contribute articles and disseminate NRA communication).

(Round 1: Your rating= 3, Average group rating= 3.88)

- ◯ 1- not a priority
- ◯ 2- low priority
- ◯ 3- medium priority
- ◯ 4- high priority
- ◯ 5- highest priority

6.6 Owners of existing websites, apps, or social media platforms regularly used by consumers or healthcare professionals to obtain medication safety information (e.g. link important NRA safety information directly through existing websites such as MiMS).

(Round 1: Your rating= 4, Average group rating= 3.98)

- ◯ 1- not a priority
- ◯ 2- low priority
- ◯ 3- medium priority
- ◯ 4- high priority
- ◯ 5- highest priority

6.7 International medication safety bodies (e.g. collaborate with the World Health Organisation, vaccine safety communication network, or other NRAs).
(New)

- ◯ 1- not a priority
- ◯ 2- low priority
- ◯ 3- medium priority
- ◯ 4- high priority
- ◯ 5- highest priority

6.8 Undergraduates or student healthcare professionals (e.g. collaborate in research).
(New)

- ◯ 1- not a priority
- ◯ 2- low priority
- ◯ 3- medium priority
- ◯ 4- high priority
- ◯ 5- highest priority

SECTION 3: General comments

Please let us know any comments you may have related to this study:

End of questionnaire.

Thank you very much.
